# A combined immunopeptidomics, proteomics, and cell surface proteomics approach to identify immunotherapy targets for diffuse intrinsic pontine glioma

**DOI:** 10.3389/fonc.2023.1192448

**Published:** 2023-08-11

**Authors:** Kirti Pandey, Stacie S. Wang, Nicole A. Mifsud, Pouya Faridi, Alexander J. Davenport, Andrew I. Webb, Jarrod J. Sandow, Rochelle Ayala, Michelle Monje, Ryan S. Cross, Sri H. Ramarathinam, Misty R. Jenkins, Anthony W. Purcell

**Affiliations:** ^1^ Department of Biochemistry and Molecular Biology and Infection and Immunity Program, Biomedicine Discovery Institute, Monash University, Clayton, VIC, Australia; ^2^ Immunology Division, The Walter and Eliza Hall Institute of Medical Research, Parkville, VIC, Australia; ^3^ Children’s Cancer Centre, Royal Children’s Hospital, Parkville, VIC, Australia; ^4^ Monash Proteomics and Metabolomics Facility, Department of Biochemistry and Molecular Biology, Monash Biomedicine Discovery Institute, Monash University, Clayton, VIC, Australia; ^5^ School of Clinical Sciences, Department of Medicine, Monash University, Clayton, VIC, Australia; ^6^ Department of Medicine, Sub-Faculty of Clinical and Molecular Medicine, Faculty of Medicine, Nursing & Health Sciences, Monash University, Clayton, VIC, Australia; ^7^ Centre for Cancer Research, Hudson Institute of Medical Research, Clayton, VIC, Australia; ^8^ Department of Molecular and Translational Medicine, School of Medicine, Nursing and Health Sciences, Monash University, Clayton, VIC, Australia; ^9^ Advanced Technology and Biology Division, The Walter and Eliza Hall Institute of Medical Research, Parkville, VIC, Australia; ^10^ Department of Neurology and Neurological Sciences and Howard Hughes Medical Institute, Stanford University, Stanford, CA, United States; ^11^ The Department of Medical Biology, The University of Melbourne, Parkville, VIC, Australia; ^12^ LaTrobe Institute for Molecular Science, LaTrobe University, Bundoora, VIC, Australia

**Keywords:** DIPG, paediatric brain cancer, proteomics, surfaceome, HLA, immunopeptidomics

## Abstract

**Introduction:**

Diffuse intrinsic pontine glioma (DIPG), recently reclassified as a subtype of diffuse midline glioma, is a highly aggressive brainstem tumor affecting children and young adults, with no cure and a median survival of only 9 months. Conventional treatments are ineffective, highlighting the need for alternative therapeutic strategies such as cellular immunotherapy. However, identifying unique and tumor-specific cell surface antigens to target with chimeric antigen receptor (CAR) or T-cell receptor (TCR) therapies is challenging.

**Methods:**

In this study, a multi-omics approach was used to interrogate patient-derived DIPG cell lines and to identify potential targets for immunotherapy.

**Results:**

Through immunopeptidomics, a range of targetable peptide antigens from cancer testis and tumor-associated antigens as well as peptides derived from human endogenous retroviral elements were identified. Proteomics analysis also revealed upregulation of potential drug targets and cell surface proteins such as Cluster of differentiation 27 (CD276) B7 homolog 3 protein (B7H3), Interleukin 13 alpha receptor 2 (IL-13Rα2), Human Epidermal Growth Factor Receptor 3 (HER2), Ephrin Type-A Receptor 2 (EphA2), and Ephrin Type-A Receptor 3 (EphA3).

**Discussion:**

The results of this study provide a valuable resource for the scientific community to accelerate immunotherapeutic approaches for DIPG. Identifying potential targets for CAR and TCR therapies could open up new avenues for treating this devastating disease.

## 1 Introduction

Brain tumors are the leading cause of cancer-related morbidity and mortality in children (1), accounting for 20% of overall cancer cases and deaths. According to the World Health Organization (WHO), primary tumors of the central nervous system (CNS) are classified from grades I to IV, reflecting varying morphology, molecular characteristics, proliferative index, response to treatment, and survival time (2, 3). Diffuse midline gliomas (DMGs) are characterized by a recurrent mutation in genes encoding histone H3 (H3K27M) and encompass heterogeneous midline locations such as the thalamus, brainstem [also called diffuse intrinsic pontine glioma (DIPG)], and spinal cord. DIPG is categorized as a grade IV glioma (2) and is spatially and temporally restricted. It arises in the ventral pons region of the brain in children as young as 3 years of age, with peak incidence observed between the ages of 6 and 9 years (4). Historically, DIPG cases were classified along with their adult glioblastoma multiforme (GBM) counterparts, but, in 2016, DIPG and other DMGs were reassigned to a separate category (2). Distinction of DIPG as a distinct disease was driven by mounting evidence that pediatric brain tumors differed from adult GBMs, not only in clinical presentation but also in their unique developmental origins (5–10) and tumor microenvironment (8), which was a direct consequence of specific driver mutations such as histone H3.3 Lys to Met (H3K27M) substitution (11–13).

Brainstem gliomas, including DIPG, account for 10%–15% of childhood CNS tumors however are the leading cause of death in children with a 5-year survival of less than 1% (14). There is no established effective chemotherapy for DIPG, and current treatments, such as radiotherapy, only alleviate symptoms temporarily but do not cure the cancer, a fact that has not changed in over four decades (15, 16). Therefore, additional and alternate approaches such as targeted immunotherapy to combat DIPG are critically needed. Despite the massive global effort to identify therapeutic targets, analysis of transcription alone is often not sufficient to determine cell surface protein abundance (17). DIPG is not as heterogeneous as cancers such as adult GBM and, therefore, is a promising candidate for targeted immunotherapies. A comprehensive antibody array screening of DIPG cultures published by Mackall and colleagues identified a panel of cell surface–expressed targets, including GD2 (18). In this study, importantly authors reported considerable overlap across DIPG cultures and conservation of cell surface–expressed markers (18). Intriguingly, this study provided the rationale for a first-in-human phase I clinical trial using chimeric antigen receptor (CAR) T cells targeting disialoganglioside (GD2), disialoganglioside, and an early recent report demonstrates promise of this approach for H3K27M-mutated DMG of the brainstem and spinal cord (NCT04196413) (19). Importantly, this trial has demonstrated that CAR T-cell immunotherapy can be delivered safely both intravenously and intracerebroventricularly (19). For pediatric patients with CNS tumors, the HER2 CAR T-cell BrainChild-01 clinical trial (NCT03500991) is recruiting, but with localization to the pons an exclusion criteria (20). Similarly, BrainChild-02 (NCT03638167) delivering EGFR806 CAR T cells has not only broad pediatric CNS tumor inclusion criteria but also exclusion criteria of diagnosis of DIPG. Intriguingly, BrainChild-03 (NCT04185038) delivering CD276 (B7H3) CAR T cells to pediatric patients with CNS tumors does include diagnosis of DIPG or DMG (21). Although these trials are ongoing, it is clear that CAR T-cell therapy has been shown to be safe with appropriate clinical precautions (19), and the cadence and route of administration are being refined for patients with DIPG (22).

Henceforth, a multi-omics approach to explore different facets of the disease and to identify therapeutic targets is an important first step in exploring additional novel treatment modalities for DIPG, probably to be applied in combination. In addition to targeting cell surface–expressed proteins, the generation of T cells that recognize cancer-specific peptides presented by human leukocyte antigen (HLA) molecules on the surface of cells is an intriguing immunotherapeutic option. The array of peptides displayed by different HLA allotypes expressed on tumor cells are collectively referred to as the immunopeptidome.

A small subset of the immunopeptidome is represented by cancer-specific peptides that may include differentially expressed tumor-associated antigens (TAAs) or peptides derived from oncogenes or gonadotrophic proteins such as cancer–testis antigens (CTAs) as well as neoantigens containing somatic mutations (23). An immunopeptidomics approach has been successfully used in several cancers including melanoma (23, 24), pancreatic ductal adenocarcinoma (25), and human papilloma virus–induced cancers (26) to eliminate tumors following vaccination or T-cell receptor (TCR) cellular therapies. Another emerging class of proteins contributing to the immunopeptidome include human endogenous retroviral (HERV)–derived antigens. HERVs are remnants of exogenous retroviruses that have integrated into the human genome during evolution (27, 28). Although transcriptionally silent, HERVs are known to become active during malignancy and are recognized as “foreign” by the immune system (27, 28) making them excellent candidates for T-cell–based immunotherapy.

There is mounting evidence that HLA-restricted peptide-based cancer immunotherapy can induce specific immune responses and impact clinical outcomes (29). Efforts are underway to develop personalized cancer vaccines for different cancers based on the immunopeptidome profiles of patients (23, 30), and, at the same time, there has been significant clinical success in the implementation of TCR therapies in solid malignancies (31, 32). Thus, in-depth characterization of the DIPG immunopeptidome will further inform immunotherapeutic approaches that harness TCR specificity and redirect their effects toward targeted malignant cells to combat the disease (33).

In this study, we have used a combination of immunopeptidomics, global proteomics, and cell surface proteomics at high resolution, to identify immunotherapy targets in patient-derived DIPG cell lines. Our in-depth immunopeptidomics analysis, for the first time, showcases the HLA class I peptide repertoire of DIPG cells. The resultant dataset was then interrogated for the presence of both TAA, CTA, and HERV-derived peptides, thereby unveiling several potential targets for T-cell–mediated immunotherapy. On the basis the global DIPG proteome data, we identified drug targets that could be used in combination with immunotherapies and new targets for the development of drugs. Finally, our in-depth cell surface proteomics identified additional CAR targets, including those identified in other cancer indications, that warrant further investigation and identified novel targets that could be used to develop novel antibody-based therapeutics.

## 2 Materials and methods

### 2.1 Primary brain sample acquisition

Primary brain (PB) control samples (non-diseased cerebellum, n = 3) were obtained by an anatomical pathologist at the Royal Children’s Hospital (Melbourne, Victoria) as part of the rapid autopsy protocol of a patient with DIPG (HREC 34049), performed within 24 h of time of death. Samples were flash-frozen and cryopreserved until further use.

### 2.2 Cell culture

Six patient-derived DIPG cell cultures (SU-DIPG27, SU-DIPG35, SU-DIPG38, SU-DIPG43, SU-DIPG58, and SF7761) were maintained in working tumor stem medium (TSM) as described previously (5). First, tumor base medium (TBM) was prepared by mixing 1:1 ratio of Neurobasal-A medium (Gibco, Thermo Fisher Scientific, USA) and Dulbecco's Modified Eagle Medium F12 (D-MEM/F-12) (Gibco), supplemented with 10 mM HEPES (N-(2-Hydroxyethyl)piperazine-N'-(2-ethanesulfonic acid)) (Gibco), 1 mM MEM sodium pyruvate (Gibco), 0.1 mM Minimal essential media (MEM) nonessential amino acids (aa) (Gibco), 1× GlutaMAX-I supplement (Gibco), and 1× Antibiotic-Antimycotic (Gibco). Second, TBM was supplemented with B27 supplement (minus Vitamin A, Thermo Fisher Scientific, USA), human epidermal growth factor (H-EGF; 20 ng/ml; Shenandoah Biotech, USA), human basic fibroblast growth factor 154 aa (H-FGF-basic 154; 20 ng/ml; Shenandoah Biotech), human platelet-derived growth factor (H-PDGF-AA; 10 ng/ml; Shenandoah Biotech), H-PDGF-BB (10 ng/ml; Shenandoah Biotech), and 0.2% Heparin solution (2 mg/mL; STEMCELL Technologies, USA).

Next-generation sequencing (NGS) for HLA class I genotyping of SU-DIPG58, SU-DIPG38, and SF7761 was performed by the Victorian Transplantation and Immunogenetics Service (West Melbourne, Victoria, Australia), whereas genotyping of SU-DIPG27, SU-DIPG35, and SU-DIPG43 was performed by PathWest Laboratory Medicine WA (Perth, Western Australia, Australia).

#### 2.2.1 Purification of HLA-peptide complexes from small cell pellets

For HLA-A*02:01 expressing DIPG cell lines (SU-DIPG27, SU-DIPG35, SU-DIPG38, and SU-DIPG43), approximately 2 × 10^7^ cells were cultured. Adherent cells were detached using Accutase (Sigma, USA), washed with Phosphate buffer saline (PBS) (Gibco, USA) before harvest by centrifugation (3,724*g*, 15 min, 4°C). Pellets were snap-frozen in liquid nitrogen and stored at −80°C until required. Small-scale immunoaffinity purifications were performed as previously described (34). Briefly, cell pellets were lysed with 100 µL of lysis buffer [0.5% Octylphenyl-polyethylene glycol (IGEPAL), 50 mM Tris (pH 8.0), 150 mM NaCl, and 1× protease inhibitor tablet (cOmplete™ Protease Inhibitor Cocktail Tablet; Roche Molecular Biochemicals, Switzerland)], gently mixed, and incubated on a roller at 4°C for 1 h. Two immunoaffinity columns were prepared using 1.2 mL of protein A sepharose (PAS, CaptivA®, Repligen, USA) incubated with 1.2 mg of BB7.2 [anti–HLA-A2 (35)] or W6/32 [pan HLA class I (36)]. Following cell lysis, samples were centrifuged at 3,724*g*, 10 min at 4°C. Lysates were first incubated with a pre-column (PAS only) for 1 h at 4°C to remove non-specific binders, followed by serial affinity capture of HLA-A*02:01 peptide complexes (BB7.2 column) and then the remaining HLA class I allotype complexes (W6/32 column). All three columns were washed with five column volumes (CVs) of 1× PBS to remove unbound antibody, detergent, and other salts. Bound peptide-HLA (pHLA) complexes were eluted using 150 µL of 10% acetic acid. To separate the proteinaceous material from peptides, the eluate was passed through a 5-kDa molecular weight cutoff filter (Amicon, Sigma-Aldrich, USA) and centrifuged at 16,060*g* for 30 min at room temperature (RT). The filtered sample was desalted using reverse-phase C_18_ stage tips (Omix, Agilent), centrifugally evaporated, and interrogated by liquid chromatography with tandem mass spectrometry (LC-MS/MS) using Q Exactive plus (QE plus, Thermo Scientific, USA).

#### 2.2.2 Purification of HLA-peptide complexes from large cell pellets

For the DIPG cell lines SF7761 and SU-DIPG58 that were more amenable to large-scale cell production, cells were cultured as described above, expanded to 2 × 10^8^ cells, harvested, and stored at −80°C until required. Purified antibodies (10 mg; GAPA3 or W6/32) were cross-linked to 1 mL of PAS resin and used to capture pHLA complexes as described (37). Briefly, frozen cell pellets were pulverized using a cryogenic mill (Retsch Mixer Mill MM 400), reconstituted in lysis buffer (refer to Section 2.2) and incubated for 45 min at 4°C with rotation. The pre-cleared supernatant was passed through a PAS pre-column, followed by sequential affinity capture with GAPA3 [anti–HLA-A3 (38)] followed by W6/32 for SU-DIPG58 or only W6/32 affinity capture for SF7761 due to the lack of appropriate allotype-specific antibodies. Bound pHLA complexes were eluted using five CVs of 10% acetic acid. Eluates were fractionated by reversed-phase high-performance liquid chromatography (RP-HPLC) on a 4.6-mm–internal diameter, 100-mm-long RP monolithic C_18_ HPLC column (Chromolith Speed Rod, Merck-Millipore, Germany) using an ÄKTA micro HPLC system (GE Healthcare) running a mobile phase consisting of buffer A (0.1% Trifluroacetic acid (TFA)) and buffer B (80% Acetonitrile (ACN)). Peptide-containing fractions were collected, concentrated using a speed vacuum concentration system (LABCONCO, USA), reconstituted in 0.1% formic acid (FA), and concatenated into eight peptide-containing pools for each antibody. Samples were analyzed by LC-MS/MS on a high-resolution Tribrid Fusion MS (Thermo Fisher Scientific, USA).

### 2.3 Identification of HLA class I bound peptides by LC-MS/MS

Both the BB7.2 and W6/32 eluates from the small-scale elution of 4 HLA-A*02:01 expressing DIPG cell lines were spiked with a mixture of 11 indexed Retention Time (iRT) peptides to aid retention time alignment (39) and acquired on a Q-Exactive plus LC-MS (Thermo Fisher Scientific, USA). Samples were acquired in data-dependent acquisition (DDA) mode using online rapid separation liquid chromatography (RSLC) nano-HPLC (Ultimate 3000 UHPLC, Thermo Fisher Scientific, USA). The samples were injected onto a 100μm, 2cm nanoviper Pepmap100 trap column prior to separation on a 75 μm × 50 cm, Pepmap100 C_18_ analytical column (Thermo Fisher Scientific, USA). The eluate was nebulized and ionized using a nano-electrospray source (Thermo Fisher Scientific, USA) with a distal coated fused silica emitter (New Objective, USA). The capillary voltage was set at 1.7 kV. The QE plus mass spectrometer was operated in the DDA mode to automatically switch between full MS scans and subsequent MS/MS acquisitions. Survey full scan MS spectra (mass to charge ratio (m/z) 375–1,600) were acquired in the Orbitrap with 70,000 resolution after accumulation of ions to a 5e5 target value with a maximum injection time of 120 ms. For MS/MS, dynamic exclusion was set to 15 s to avoid resequencing the same analyte. The 12 most intense charged ions (z ≥ +2) were sequentially isolated and fragmented in the collision cell by higher-energy collisional dissociation (HCD) with a fixed injection time of 120 ms, 35,000 resolution, and automatic gain control (AGC) target of 2e5 with isolation window of 1.8 *m*/*z*, intensity threshold 1.7e4, and scan range of 200–2,000 *m*/*z*.

The eight peptide fractions derived from each of the large cell pellets of SF7761 and SU-DIPG58 cell lines were analyzed on a Tribrid Fusion LC-MS (Thermo Fisher Scientific, USA) with addition of the iRT peptide mix. Peptides were loaded onto a PepMap Acclaim 100 C_18_ trap column (5 µm particle size, 100 µm × 2 cm and 100Å (Thermo Fisher Scientific, USA) at 15 µL/min using an Ultimate 3000 RSLC nano-HPLC (Thermo Fisher Scientific, USA). Peptides were eluted and separated at a flow rate of 250 nL/min on an in-line analytical column PepMap RSLC C_18_, 2 μm particle size, 75 μm × 50 cm, and 100 Å, (Thermo Fisher Scientific, USA) using 125-min linear gradient as described previously (37). Peptides were introduced using nano–electrospray ionization method into the Orbitrap Fusion MS at a source temperature of 275°C. All MS spectra (MS1) profiles were recorded from full ion scan mode 375–1,800 *m*/*z*, in the Orbitrap at 120,000 resolution with AGC target of 400,000 and dynamic exclusion of 15 s. The top 12 precursor ions were selected using top speed mode at a cycle time of 2 s. For MS/MS, a decision tree was made to aid selecting peptides of charge state +1 and +2–6 separately. For singly charged analytes, only ions falling within the range of *m*/*z* 800–1,800 were selected, whereas, for +2 to +6 *m*/*z*, no such parameter was set. The C-trap was loaded with a target of 200,000 ions with an accumulation time of 120 ms and isolation width of 1.2 amu. Normalized collision energy was set to 32 HCD, and fragments were analyzed in the Orbitrap at 30,000 resolution.

### 2.4 Mass spectrometry data analysis for immunopeptidomics samples

Immunopeptidomics MS/MS raw files generated on the Tribrid Fusion and QE Plus were exported and analyzed using Peaks 8.5 software (Bioinformatics Solutions Inc.) searching against the human proteome (Uniprot, accessed 15 June 2017; 20,182 entries). The following search parameters were used: error tolerance of 10 ppm using monoisotopic mass for precursor ions and 0.02Da tolerance for fragment ions; enzyme used was set to none with following variable modifications: oxidation at Met; deamidation at Asp and Gln; and phosphorylation at Ser, Thr, and Tyr. False discovery rate (FDR) was estimated using a decoy fusion method (40). For data processing, first, a 5% FDR cutoff was applied. Peptides matched to decoy database, iRT peptides, duplicates, and those detected in blanks were removed. Second, only peptides with 8–12 aa were retained with modified versions of the peptides also removed.

### 2.5 Peptide binding motif and protein pathway analyses

The binding motif of HLA peptides was interpreted using IceLogo (41) and binding affinity to different HLA alleles ascertained using NetMHC pan 4.0 RRID : SCR_021651 (42). Peptides were identified as either strong binders (SBs; based on rank threshold of 0 to 0.5) or weak binders (WBs; based on rank threshold of 0.5 to 2.0). Peptides identified were cross-referenced with Immune Epitope Database and Analysis Resource (IEDB) that lists all HLA-restricted peptides identified in humans (43). Whereas, to identify brain-specific proteins present in the data, Human Brain Protein Atlas (HBPA) (44) was used. The data were plotted in GraphPad Prism version 9.1 (GraphPad Software, USA, www.graphpad.com, RRID : SCR_002798).

### 2.6 Proteomic sample preparation

Proteomics was performed on pHLA depleted cell lysates (Sections 2.1 and 2.3; n = 5) as well as part of primary non-diseased brain sample controls (cerebellum, n = 3) obtained from rapid autopsies of patients with DIPG (as described in Section 2.2), using sodium dodecyl sulfate (SDS)–based suspension trapping (S-TRAP) method. Briefly, lysates were thawed, vortexed, and centrifuged at 3,978*g* for 10 min at RT. Protein concentration was measured using Direct Detect Spectrometer (Millipore, USA). A volume equivalent to 400 µg of protein was mixed with an equal volume of 2× lysis buffer [10% SDS, 100 mM triethylammonium bicarbonate (TEAB; Sigma), pH 8.5]. After lysis, 20 mM (final concentration) of Tris(2-carboxyethyl) phosphine hydrochloride (TCEP; Sigma) was added to reduce the samples by incubation at 60°C for 15 min. For alkylation, 20 mM (final concentration) of iodoacetamide (IAA; Sigma) was added, and samples were incubated in the dark for 15 min at RT. Samples were then acidified using 1.2% phosphoric acid. Colloids were generated by adding S-TRAP binding buffer (90% methanol in 100 mM TEAB) in a 1:7 ratio of buffer to starting material. The resulting colloid samples were loaded onto the S-TRAP filter (Profiti, USA) and centrifuged at 3,978*g* for 30 s at RT. Columns were washed thrice using the binding buffer. Protein digestion in the S-TRAP filter was performed by using Trypsin (Sigma, USA) made in digestion buffer (50 mM TEAB) at a 1:60 ratio of enzyme to sample. Tubes were sealed and incubated at 37°C overnight. For peptide recovery, S-TRAP filters were washed sequentially with 80 µL of 50 mM TEAB followed by 80 µL of 0.1% FA and, finally, 80 µL of 50% ACN with 0.2% FA. Samples were concentrated using a speed vacuum concentration system (LABCONCO, USA) and reconstituted in 20 µL of 0.1% FA.

### 2.7 Proteomics data acquisition

Proteomics samples were diluted 1:10 in 0.1% aqueous FA and spiked with iRT peptide mix. Samples were acquired using a hybrid trapped ion mobility (TIMS)-quadrupole time-of-flight (TOF) MS (Bruker TimsTOF pro, Bruker Daltonics, Germany) coupled to a nanoElute liquid chromatography system. The sample (1-2 µL) was loaded directly onto the C_18_ analytical column (Aurora, IonOpticks) of 1.6 μm particle size and 75 μm × 25 cm and 120Å using a 20-min linear gradient of buffer A1 (2% ACN and 0.1% FA). Peptides were eluted from the column with flow rate set to 400 nL/min, by a linear step-wise gradient of buffer B1 (100% ACN and 0.1% FA) against buffer A1 initially to 17% over 60 min, then to 25% over 30 min, and 37% over 10 min followed by rapid rise to 95% over 10 min. DDA was performed with following settings: *m/z* range, 100–1,700 *m/z*; capillary voltage, 1,600 V; target intensity, 30,000; and TIMS ramp, 0.60 to 1.60 Vs/cm^2^ for 166 ms. All SU-DIPG cell lines and SF7761 were acquired as technical replicates (n = 3), whereas PB samples were acquired as biological replicates from the same patient (n = 3).

For proteomics data analysis, the. TDF files generated on the Bruker TimsTOF for the samples were searched using MaxQuant computational proteomics platform version 2.0 (45). The following search parameters were used: N-terminal acetylation along with oxidation at Met and deamidation at Asp and Gln was set as variable modifications. Cys carbamidomethylation was set as a fixed modification. Bruker TIMS was selected under instruments with 20 ppm as first search peptide tolerance and 10 ppm as main search peptide tolerance. The enzyme specificity was set to Trypsin with a maximum of two missed cleavages being chosen. Quantification in MaxQuant was performed using the built-in extracted ion chromatogram-based label-free quantification (LFQ) (46). The spectra were searched by the Andromeda search engine against the human proteome (Uniprot, accessed 15 June 2017; 20,182 entries) including contaminants. Possible sequence matches were restricted to 8 to 25 aa, a maximum peptide mass of 4,600 Da. A FDR of 0.01% was set for proteomic analysis. Protein identification required at least one unique or razor peptide per protein group.

Further downstream analysis of the data was performed on the basis of LFQ values using Perseus software (version 1.4.0.6) (45). LFQ values were log2-transformed and filtered on the basis of valid values found in at least three samples in any group. Missing values were replaced from normal distribution. To identify proteins being significantly regulated in DIPG cell lines compared to PB, a two-sided t-test was performed at 0.01% FDR. For visualization in the form of heatmap, log2-transformed scores were normalized by Z-score. The −logP value was converted into −log10 P-value, and volcano plots was made using ggVolcanoR app (47) by plotting −log10 P-value vs. the log2 fold change difference.

Specific databases were used to annotate the proteomics data. To identify proteins expressed on the surface of cells, Cell Surface Protein Atlas (CSPA) (48) was used. To identify proteins that are targets of Food and Drug Administration (FDA)–approved drugs, we used the Human Protein Atlas (HPA) druggable proteome https://www.proteinatlas.org/humanproteome/tissue/druggable) (44). All databases were added under annotation in Perseus and used to filter rows.

Protein network analyses were performed using Cytoscape (version 3.7.2) RRID : SCR_003032 (49) plug-in ClueGO version 2.5.7 (50). The enrichment of proteins was determined by right-sided hypergeometric test with Bonferroni step down correction and kappa score threshold of 0.4.

### 2.8 Cell surfaceome sample preparation

Patient-derived SU-DIPG cell lines SU-DIPG 4, SU-DIPG 13, SU-DIPG 17, SU-DIPG 19, SU-DIPG 21, SU-DIPG 25, and SU-DIPG 33 were analyzed using cell surface proteomics in quadruplicate, using an amino-oxy-biotin labeling technique (51). Briefly, cells were labeled for surface sialic acid residue by biotinylating with 1 mM sodium meta-periodate (Thermo, cat. no. 20504), 200 μM amino-oxy-biotin (Biotium, cat. no. 90113), and 10 mM aniline (Sigma, stock no. 10.97 M) in PBS (pH 6.7) for 1 h at 4°C. Following this, the reaction was quenched with glycerol, cells were washed twice, and a small aliquot taken to test the efficiency of biotinylation, using a streptavidin-labeled fluorescent marker. The cells were then resuspended in lysis buffer [1% v/v Igepal CA-630, 150 mM NaCl, 1× protease inhibitor (complete, without Ethylenediamine tetraacetic acid (EDTA), Roche), 5 mM IAA, and 10 mM Tris-HCl (pH 7.6)]. Nuclei were removed by centrifugation at 2,800*g* for 10 min, at 4°C. The remaining biotinylated proteins in the lysate were enriched by incubation with high-capacity streptavidin-agarose (Pierce, Country) for 60 min at 4°C. After three washes with lysis buffer and PBS/0.5% (w/v) SDS, samples were incubated at RT for 20 min with 100 mM Dithiothreitol (DTT). Further washes with urea buffer [6 M urea and 100 mM Tris-HCl (pH 8.5)] were followed by a final incubation with urea buffer containing 50 mM IAA in the dark for 20 min. Subsequently washes were performed with urea buffer, PBS, and then water. The proteins were digested on-bead overnight in 100 µL of 50 mM ammonium bicarbonate containing 2 µg of Trypsin Gold. The following morning, peptides were collected by centrifugation, and tryptic fractions were analyzed for mass spectrometry analysis.

After sample processing, peptides were resuspended in 2% ACN/1% FA and separated by reversed-phase liquid chromatography on a M-class UHPLC system (Waters, USA) using a 250 mm × 75 μm column (1.6 µm C18, packed emitter tip; Ion Opticks, Australia) with a linear 90-min gradient at a flow rate of 400 nL/min from 98% solvent A (0.1% FA in Milli-Q water) to 35% solvent B (0.1% FA and 99.9% acetonitrile). The nano-UPLC was coupled on-line to an Impact II mass spectrometer equipped with a CaptiveSpray ionization source (Bruker Daltonics, Germany) and column-oven at 40°C (Sonation, Germany). The Impact II was operated in a data-dependent mode using a 1.5-s cycle time, switching automatically between one full-scan at 4 Hz and subsequent MS/MS scans for the remaining time with spectra rate determined using peptide intensity. The instrument was controlled using otofControl version 4 (Bruker).

Raw files were analyzed using MaxQuant (version 1.5.8.3). The database search was performed using the Uniprot Homo sapiens database (downloaded October 2020) plus common contaminants with strict trypsin specificity allowing up to two missed cleavages. The minimum peptide length was 7 aa. Carbamidomethylation of cysteine was a fixed modification, whereas N-acetylation of proteins N-termini and oxidation of methionine were set as variable modifications. During the MaxQuant main search, precursor ion mass error tolerance was set to 0.006 Da. PSM and protein identifications were filtered using a target-decoy approach at an FDR of 1% with the match between runs option enabled.

Downstream analysis of the data was performed using Perseus as mentioned in Section 2.8. Identification of proteins with transmembrane helices was performed using prediction server TMHMM (52, 53) (https://services.healthtech.dtu.dk/service.php?TMHMM-2.0) based on the PredHel (number of predicted helices present in the protein) score of 3 or more.

### 2.9 Validation of cell surface protein expression in DIPG cell lines by flow cytometry

Cell surface HLA class I expression for the cell lines was measured by flow cytometry following surface staining using hybridoma supernatants for anti–HLA-A2 {BB7.2 [HB-82; American Type Culture Collection (ATCC)] (35); produced in-house}, anti–HLA-A3 [GAPA3 (HB-122; ATCC) (38); produced in-house], and pan HLA class I [W6/32 (HB-95; ATCC) (54); produced in-house] as primary antibodies followed by a goat anti-mouse Immunoglobulin G Phycoerythrin (IgG PE) secondary antibody [1:250 dilution (cat. no. 1030-09); Southern Biotech, cat. no. 1030-09, RRID : AB_2794297, USA] using a LSRII flow cytometer.

Cell surface protein targets were validated using antibody labeling of DIPG cell lines using flow cytometry. Briefly, single-cell suspensions of DIPG cells were antibody labeled with anti-CD276-PE (clone MIH42, BioLegend), anti-CD47-BV421 (clone CC2C6, BioLegend), or anti-CD63-APC (clone H5C6, BioLegend) (200 µL), and data were acquired on a Fortessa XX-20 with Diva software. Analysis of the flow cytometry data was performed using FlowJo software RRID : SCR_008520 (version 10.2, BD Biosciences, USA).

## 3 Results

### 3.1 Profiling HLA class I ligands derived from pediatric DIPG cell lines

**Figure 1:**
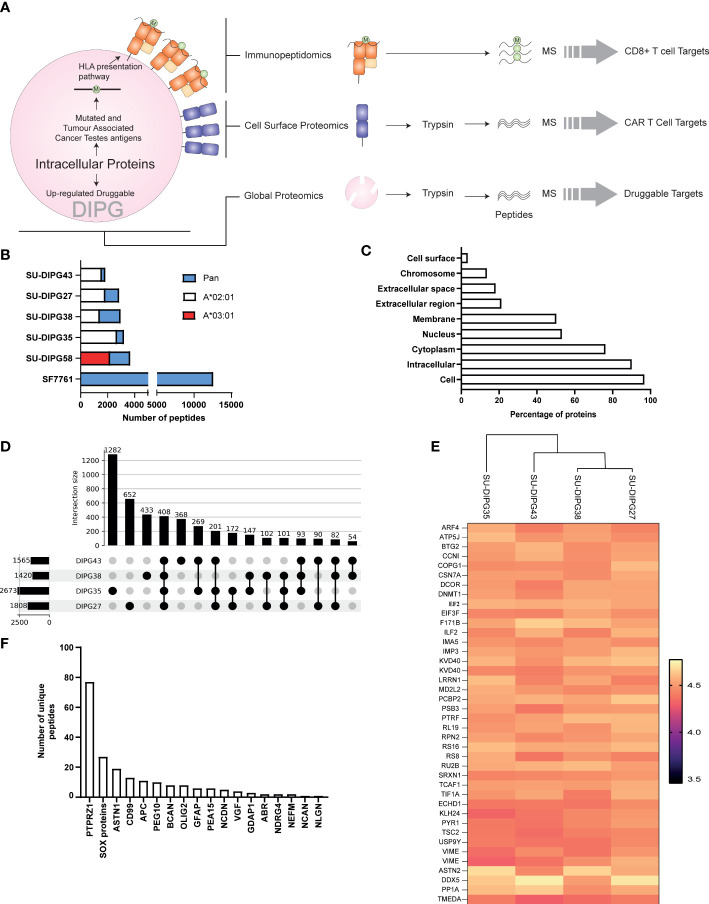
The immunopeptidome of SU-DIPG and SF7761 cell lines. **(A)** A three-pronged approach was taken to identify intracellular (global proteomics), CD8^+^ immune visible (immunopeptidomics), and CAR T-cell (surface expressed) targets of DIPG. **(B)** Distribution of allele specific and pan class I peptides identified across DIPG cell lines, wherein white represents HLA-A*02:01 peptides, red represents HLA-A*03:01 peptides, and blue represents number of pan class I allotype bound peptides identified. **(C)** GO slim ontology depicting different cellular compartments for immunopeptidome source proteins. **(D)** Upset plot depicting unique and shared HLA-A2 class I peptides between SU-DIPG27, SU-DIPG35, SU-DIPG38, and SU-DIPG43. **(E)** Heatmap of top 40 conserved shared peptides across the four DIPG HLA-A2 cell lines. **(F)** A refined subset of brain-specific proteins identified in DIPG cell lines identified in the immunopeptidome using Human Brain Protein Atlas.

We characterized the HLA class I peptide repertoire of six DIPG patient-derived cell cultures (SU-DIPG27, SU-DIPG35, SU-DIPG38, SU-DIPG4, SU-DIPG58, and SF7761) using immunopeptidomics (55) (**Figure 1A**). To our knowledge, this represents the first detailed description of the immunopeptidome of DIPG. Prior to undertaking the antigen discovery approach, HLA genotyping was performed for all six cell lines revealing a total of 22 different HLA class I allotypes (**Supplementary Table 1**). There were several shared allotypes among the cell lines, including HLA-A*02:01 (n = 4), A*01:01 (n = 2), A*68:01 (n = 2), B*44:02 (n = 2), and C*04:01 (n = 2). We assessed the level of HLA class I cell surface expression in the cell lines using flow cytometry. Allotype-specific antibodies such as BB7.2 (anti–HLA-A2) was used for SU-DIPG27, SU-DIPG35, SU-DIPG38, and SU-DIPG43 and GAPA3 (anti–HLA-A3) for SU-DIPG58. SU-DIPG35 demonstrated the highest detectable HLA-A2, with all cell lines showing different levels of HLA-A2 expression (**Supplementary Figure 1A**). W6/32 (anti-pan class I antibody) was used to stain for remaining HLA class I alleles across all six cell lines. As expected, HLA class I expression varied among different cell lines with the highest expression observed for SF7761 (**Supplementary Figure 1B**).

A total of 26,116 peptides were identified across all cell lines using LC-MS/MS (**Supplementary Table 1**), of which 20,536 (**Supplementary File 1A**) were found to be non-redundant peptides of 8–12 aa in length. For the four HLA-A2^+^ cell lines, using an HLA-A2–specific antibody (BB7.2) for immunoprecipitation of HLA class I complexes resulted in identification of 7,459 HLA-A*02:01–restricted peptides (**Supplementary Table 1**), non-redundant peptides are listed in **Supplementary File 1B** with the highest number being identified in SU-DIPG35 (2,671 peptides) and the lowest in SU-DIPG38 (1,418 peptides) (**Figure 1B**). All the HLA-A2 bound peptides followed canonical length distribution for HLA class I ligands, with the majority of peptides (~63%–70%) being nonamers (9 mers) (**Supplementary Figure 2A**). As expected, we observed adherence to the HLA-A*02:01 consensus binding motif for the 9-mer peptides across all cell lines, with Leu/Met at position 2 (P2) and Leu/Val at P9 (**Supplementary Figures 2B–E**). Similarly, for SU-DIPG58, the only HLA-A3^+^ cell line, 2,115 restricted peptides were identified using the allotype-specific GAPA3 antibody to immunopreciptate the HLA-A3 peptide complexes (**Figure 1B**, **Supplementary Table 1**). The HLA-A3 peptides followed canonical HLA class I length distribution (**Supplementary Figure 2A**) and exhibited the canonical HLA-A3 binding motif (**Supplementary Figure 2F**) with Lys/Arg at P1 and P9 anchor positions.

In addition to HLA-A2 and HLA-A3 peptides, 16,542 peptides were identified in the pan HLA class I (W6/32) immunoprecipitations from the six DIPG cell lines, with the highest number (11,582) of peptides identified from SF7761 cells (**Figure 1B**, **Supplementary Table 1**). Among the peptides identified using W6/32 immunoprecipitation, 9 mers represented 46%–57% of the entire peptide repertoire, followed by 10 mers with 14%–23% and 11 mers with 7%–15% (**Supplementary Figure 3**). To assign HLA restriction to the peptides identified using the pan class I antibody, their binding affinities for the relevant HLA allotypes were ranked using NetMHC 4.0 (42). All HLA allotypes were assigned peptide ligands predicted to be both SBs and WBs, which were collectively used to plot the binding motif for each allele using Icelogo (**Supplementary Figures 4A–F**).

### 3.2 Identification of brain-specific peptides in the immunopeptidome

The source protein landscape of DIPG cell lines was explored to gain insight into the class of proteins contributing toward the immunopeptidome. HLA class I presented peptides that were derived from a total of 6,312 non-redundant source proteins; most of the proteins contributing to HLA class I peptides came from cytoplasmic proteins followed by nucleus and membrane proteins (**Figure 1C**). Further analysis of the immunopeptidome revealed that, in all cell lines (regardless of antibody used for immunopeptidomics), 69% to 89% of the source proteins contributed to only one peptide being presented at the cell surface (**Supplementary Figures 5A, B**).

Comparative analysis between the four HLA-A*02:01–positive SU-DIPG lines showed that there were 408 peptides shared (**Figure 1D**, **Supplementary File 1C**) across them. On the basis of their log2-transformed intensity observed in immunopeptidomics data, the top 40 peptides were selected, and a heatmap was plotted. As expected, some of the peptides were derived from the most abundant proteins involved in transcription (ribosomal proteins RS8, RS16, and RL19), translation (elongation and initiation factors), and filament protein such as Vimentin (**Figure 1E**).

Next, the immunopeptidomics data were cross-referenced with the HBPA database to identify peptides presented from brain-enriched proteins. A total of 1,783 brain-enriched peptides, restricted to different HLA class I allotypes, were identified (**Supplementary File 2A**). This included proteins such as neurofilament medium (NEFM) neurochondrin (NCDN), neurocan core protein (NCAN), and neuroligin-3 (NLGN3) and brevican core protein (BCAN). Of the total 1,783 peptides identified, we observed a high contribution derived from PTPRZ1 protein (77 peptides), as well as SOX proteins, ASTN1, and CD99 (**Figure 1F**). Notably, four peptides SILDIVTKV (RFTN2), AIIDGVESV (PTPRZ1), KVFAGIPTV (PTPRZ1), and NLDTLMTYV (NLGN4X) (**Supplementary Table 2**) were also reported as immunogenic in patients with GBM providing strong evidence that these peptides should also be explored in patients with HLA-A2^+^ DIPG (29, 56). Furthermore, we identified eight peptides from PEG10 (with two peptides restricted to HLA-A*02:01) and two peptides from PEG3 proteins (**Table 1**). Both proteins are potentially oncogenic HERV (57). Of the 1,783 peptides identified, 1,082 peptides were found previously reported in the publicly available IEDB database that may be of therapeutic significance (**Supplementary File 2B**). Additional interrogation of publicly available IEDB T-cell data revealed that, among the peptides derived from brain-specific proteins, there were 25 known CD8^+^ T-cell epitopes (**Supplementary File 2C**).

**Table 1:** Peptides from oncogenes, testis antigen, and HERVs identified in DIPG cell lines.

Protein name	Peptide sequence	HLA restriction	Database
ABR	RLYPAFMEGI*, FTWEGLYNV*	A*02:01	HBPA
BCAN	FLGDPPEKL	A*02:01	HBPA
BRAF	YLSPDLSKV*, KIGDFGLATV*	A*02:01	TAA
CD99	AVQRTLLEK*, AISSFIAYQK*	A*03:01	HBPA
CSPG4	TLAPPLLRV*	A*02:01	TAA
EphB1	ESTSLVIAR	A*68:01	HBPA
IL13Ra	IVDPGYLGY*	A*01:01	CTA
MAGED4	MNIGDEALIGR	A*02:01	CTA
NCDN	KEAEPDLLAVL*, SLLKEPQKVQL* RLLSTSPAL*	A*02:01	HBPA
NPTX2	ALLQRVTEL	A*02:01	HBPA
NRXN3	TLHSVFFTL, VADPVTFKS	A*02:01	HBPA
OLIG2	SLPGSGLPSV	A*02:01	HBPA
PTPRNZ1	MIWEHNVEV, TQDDYVLEV*	A*02:01	HBPA, CSPA
PEG10	AQNGIPLRI*, SIPSGHVYSL*	A*02:01	HBPA
PRAME	SLLQHLIGL*	A*02:01	CTA
SOX8/SOX9/ SOX10	KLADQYPHL*	A*02:01	TAA

* Seventeen peptides have been previously reported in IEDB.

### 3.3 Mining the DIPG immunopeptidomes for cancer-specific antigens

**Figure 2:**
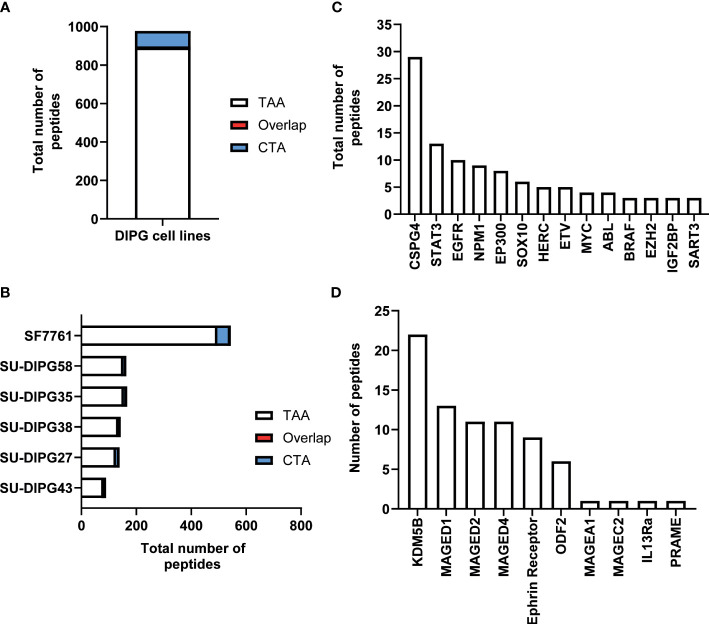
An overview of TAA and CTA peptide candidates identified in DIPG. **(A)** Bar graph representing total number of unique TAAs, CTAs, and overlapping peptides identified across six DIPG cell lines. **(B)** Bar graph depicting total number of cancer-specific peptides identified in each DIPG cell line. A representation of total number of peptides identified from selected **(C)** oncogenic proteins from TANTIGEN and **(D)** CTAs from CTdatabase.

The immunopeptidome data from all six DIPG cell lines were further interrogated for the presence of known TAAs and CTAs using two different databases: Tumor T-cell Antigen Database (TANTIGEN; http://cvc.dfci.harvard.edu/tantigen) (58) and CTdatabase (http://www.cta.lncc.br/) (59). Within the combined DIPG patient cell line dataset, a total of 977 non-redundant cancer associated peptides were identified, with 891 contained within the TANTIGEN database (**Supplementary File 3**), 80 from the CTdatabase (**Supplementary File 4A**), and six overlapping peptides (**Supplementary File 4B**) that were present in both databases (**Figure 2A**). Varying numbers of TAAs were identified across all DIPG cell lines with the most peptides (516) identified in SF7761 (**Figure 2B**). Across the entire dataset, 19 peptides have been previously reported as T-cell epitopes in the TANTIGEN and CTdatabase (**Supplementary Table 2**) in ovarian (60) and renal (61, 62) cancers, of which 13 peptides are restricted to HLA-A2. Two peptides KIQEILTQV (IGF2BP3) and TMLARLASA (CSPG4) have been reported immunogenic in patients with GBM (56) (**Supplementary Table 2**). Some other source proteins contributing to the highest number of TAA peptides included CSPG4 (31 peptides), NPM1 (14 peptides), and SOX proteins (11 peptides) (**Figure 2C**).

Furthermore, the investigation of source proteins contributing to CTA peptides revealed several interesting candidates. The source protein for peptide IVDPGYLGY was interleukin-13 receptor subunit alpha-2 (IL13Rα2), which has been reported to be overexpressed in 85% of DIPG cases (63) and an effective CAR T-cell target for glioblastoma tumor regression (64). Other peptides of interest included peptides previously reported as immunogenic and derived from PReferentially expressed Antigen in MElanoma (PRAME), Melanoma associated antigen A1 (MAGEA1), and Ephrin receptors proteins (**Table 1**, **Figure 2D**).

### 3.4 Validation of potential DIPG epitopes

**Figure 3:**
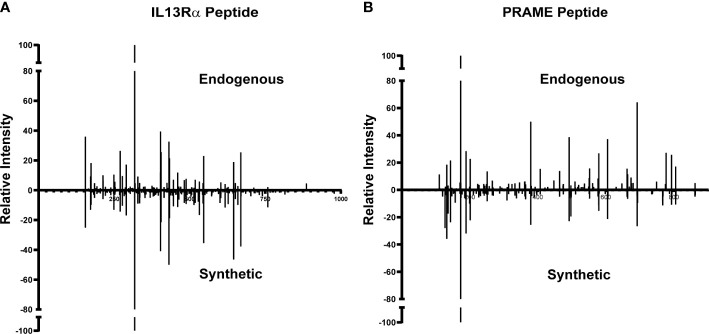
Validation of TAA and CTA peptide candidates using synthetic peptides. Similar MS/MS fragmentation spectra of endogenous and synthetic peptide for **(A)** IVDPGYLGY (IL13Rα2) and **(B)** SLLQHLIGL (PRAME).

Of the 977 total CTA- and TAA-derived potential immunogenic peptides identified, 12 peptides from proteins including IL13Rα2, PRAME, and MAGE family were selected (**Table 2**) and validated by comparing their LC-MS/MS characteristics to their synthetic counterparts. Eleven comparative synthetic and endogenous peptide MS/MS fragmentation patterns matched with high Pearson correlation values ranging from 0.92 to 0.99 (**Supplementary Figure 6**) with a difference in retention time of ±3 min. Representative mirror plots for two peptides of interest from proteins IVDPGYLGY (IL13Rα2) and SLLQHLIGL (PRAME) solidify the data outlined above (**Figure 3**). This dataset provides an ideal starting point for pHLA targeting therapeutics for patients with DIPG.

**Table 2:** Correlation of experimental and synthetic peptides for candidate immunotherapeutic proteins.

Protein	Peptide sequence	Pearson correlation R value	RT in experimental	RT in synthetic
IL13Rα	IVDPGYLGY	0.9235	73.9	75.8
PRAME	SLLQHLIGL	0.9728	74.9	75.8
MAGE A1	KVLEYVIKV	0.9569	50.6	52.7
MAGE C1	FAFGEPREL	0.9874	54.2	54.1
MAGE D1	KEIDKEEHL^*^	0.9849	21.4	19.9
MAGE D2	NADPQAVTM	0.9775	26.6	28.8
	DVYPEIIER^#^	0.9742	52.5	55.6
	YSLEKVFGI	0.5359	68.2	57.4
	KEIDKNDHLYIL	0.9607	43.0	46.8
MAGE F1	FLFGYPKRL	0.9564	55.8	58.9
	ILFPDIIARA	0.9936	71.8	70.1
MAGE D4	MNIGDEALIGR	0.9828	44.3	46.1

* Also identified in MAGE D4 protein. # Also identified in MAGE D1 protein.

### 3.5 The proteomic landscape of DIPG cell lines

**Figure 4:**
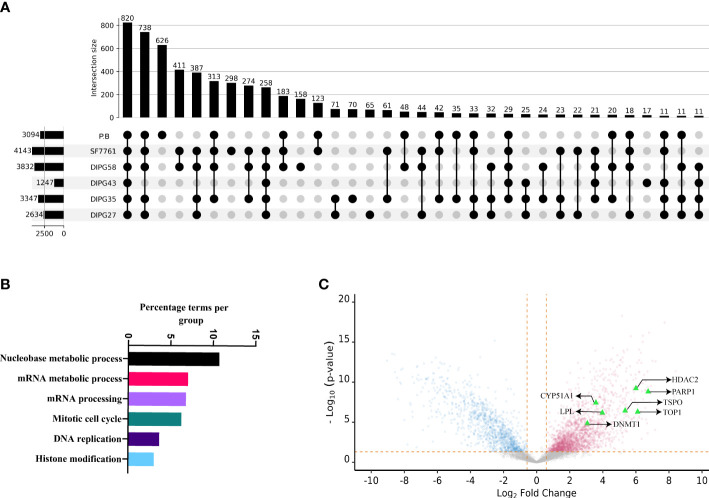
The proteomic landscape of DIPG cell lines compared to primary brain samples. **(A)** Upset plot depicting overlap between proteins identified in DIPG cell lines and PB samples. **(B)** Gene ontology/pathway analysis of proteins shared between DIPG cell lines and not PB. **(C)** Volcano plot depicting log2 fold change and corresponding −log10 adjusted p-value of all differentially expressed proteins in DIPG cell lines (pink) when compared to PB controls (steel blue), with highlighted proteins (green triangles) known targets of FDA-approved drugs.

To understand the systemic dysregulation occurring and driving DIPG, protein expression and patient-derived DIPG cell cultures (SU-DIPG27, SU-DIPG35, SU-DIPG43, SU-DIPG58, and SF7761) were investigated and compared to non-diseased PB. Whole proteomics was performed on cell lysate depleted of pHLA using S-TRAP technique followed by enzymatic digestion with trypsin and LC-MS/MS analysis. A total of 6,947 proteins were identified across the entire dataset at 1% FDR (cell lines and PB samples). The number of proteins identified per sample ranged between 1,247 and 4,143 (**Figure 4A**, **Supplementary Files 5A–F**). Comparative analysis between DIPG cell lines and brain samples showed that 820 proteins are shared between all samples, whereas 626 proteins were only identified in non-diseased PB and not detected in DIPG cells (**Figure 4A**). Pathway analysis of the 820 shared proteins was performed using the Cytoscape plug-in ClueGO (50). Importantly, pathways related to axon and synapse development were found to be significantly enriched against the background GO term datasets (**Supplementary Figure 7**), reflecting the neuronal origin of the DIPG cell lines and reiterating their relevance for studying pediatric brain tumors.

Furthermore, the peak areas expressed as LFQ values (maxLFQ) for proteins were transformed and filtered resulting in robust quantification of 4,996 proteins across the dataset (**Supplementary File 6A**). To identify proteins enriched in DIPG cell lines compared to PB, the transformed LFQ values were used, and Student’s t-test was performed using Perseus software. On the basis the test, the log fold change and P-value were used to make volcano plot depicting proteins enriched in DIPG cell lines. Of the 4,996 proteins, a total of 2,025 proteins were found to have higher expression in DIPG cell lines (**Supplementary File 6B**). Notably, analysis of the DIPG enriched proteins performed using GO terms revealed that majority of them were involved in pathways related to nucleobase metabolism, mRNA synthesis and processing, cell cycle, and histone modification (**Figure 4B**). Proteins that had the highest log fold change in DIPG cell lines were Chromodomain Helicase DNA Binding Protein 4 (CHD4), Flap endonuclease 1 (FEN1), DEAD-box helicase 46 (DDX46), X-Ray Repair Cross Complementing 6 (XRCC6), Non-POU domain containing octamer binding (NONO), and Serine Hydroxymethyltransferase 2 (SHMT2) (**Supplementary Figure 8**). These proteins are known chromatin remodelers and play a role in DNA repair after damage.

In addition, histone proteins (H2A, H2B, and H3) were found to be more abundantly expressed in the DIPG cell lines, along with proteins involved in the antigen processing and presentation pathway including HLA-A, HLA-B, and HLA-C along with TAP1, TAP2, TAP binding protein (TABBP) and proteasome activator and regulatory subunits such as Proteasome Activator Subunit 1 (PSME3), 26S proteasome non-ATPase regulatory subunit 7 (PSMD7), and 26S proteasome non-ATPase regulatory subunit 11 (PSMD11) (**Supplementary Figure 8**).

To identify cell surface proteins, we cross-referenced our data with the CSPA database and found a total of 273 (18%) nominal cell surface proteins enriched in our DIPG dataset (**Supplementary File 6C**). This included cluster of differentiation (CD) markers along with extracellular matrix (ECM) proteins such as Integrins and Fibronectin (FN1). Next, we sought to identify proteins or pathways in DIPG that could be a potential drug target that we used as HPA druggable proteome (a list that comprises of 812 proteins that are targets of current FDA-approved drugs). Of the 2,025 proteins enriched in DIPG cell lines, we identified 44 proteins that could be potential targets of various drugs (**Supplementary File 6D**). As expected, some of the proteins identified included known drug targets such as histone deacetylases (HDAC1 and HDAC2), cyclin dependent kinases (CDK4 and CDK6), and other proteins like Poly(ADP-Ribose) Polymerase 1 (PARP1) and DNA (cytosine-5)-methyltransferase 1 (DNMT1). Another interesting target identified was the TSPO -(Translocator protein) that is known to be expressed exclusively in brain cancers, including GBM (65), and can be used for delivering drugs across the blood brain barrier (66) along with DNA topoisomerase I (TOP1), Lipoprotein Lipase (LPL), and DNMT1 (**Figure 4C**). Some other proteins of interest include the surface protein FN1 and the enzyme butyrylcholinesterase (BCHE), both being known as unfavorable prognostic markers for different cancers (67–69) along with EphA2.

### 3.6 Identifying DIPG cell surface proteins as therapeutic targets

**Figure 5:**
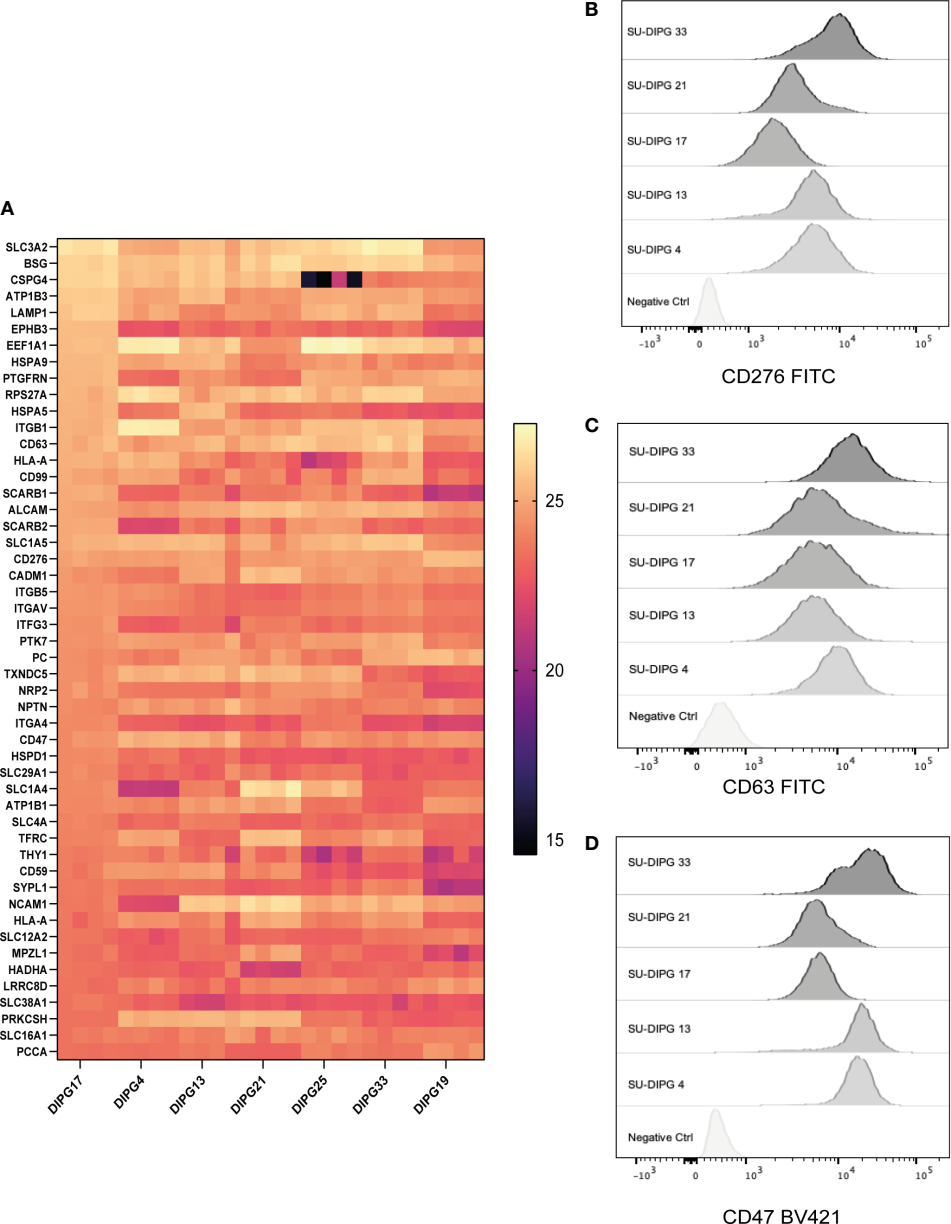
The surfaceome of DIPG cell lines and validation of cell surface expression of surface proteins. A total of 3,033 surface-expressed proteins were identified across the seven DIPG cell lines. **(A)** The heatmap is a representation of the top 50 proteins ranked by their log-transformed LFQ intensities (complete list in **Supplementary File 7**). Flow cytometry validation of **(B)** CD276, **(C)** CD63, and **(D)** CD47 proteins identified using cell surface proteomics, which can be potential target antigens.

Immunotherapies such as bispecific antibodies and CAR T cells require the identification of cancer-specific cell surface proteins. Therefore, mapping the DIPG cell surface proteome allowed the identification of potential and novel CAR targets. To our knowledge, this represents the first detailed description of the surfaceome of DIPG. To capture DIPG cell surface proteins, we biotinylated surface glycoproteins with amino-oxy-biotin to facilitate streptavidin pulldown and identification by LC-MS/MS. Seven DIPG cell lines, namely, SU-DIPG4, SU-DIPG13, SU-DIPG17, SU-DIPG18, SU-DIPG21, SU-DIPG25, and SU-DIPG33, were processed for cell surface protein identification (**Figure 1A**). A total of 3,033 proteins were found to be expressed on the surface of 7 SU-DIPG cell lines (**Supplementary File 7A**). Of the 3,033-proteins, a total of 799 shared proteins were detected across all seven cell lines (**Supplementary File 7B**). The top 50 cell surface proteins based on spectral intensity identified using this approach included proteins belonging to family of solute carrier (SLC) such as SLC3A2, SLC1A5, SLC16A1, SLC1A4, SLC6A8, SLC12A2, and SLC29A1 proteins along with several CD markers (**Figure 5A**). When cross-referenced with CSPA, 421 proteins were identified (**Supplementary File 7C**), which included nine CD markers including CD276 (B7-H3), CD47, and CD63. Some other targets of interest include EGFR protein, Human epidermal growth factor receptor 3 (HER3), and Ephrin proteins (from both EphA and EphB) along with IL13αR.

CD276 is an immune checkpoint protein that is expressed at a high level across all seven DIPG cell lines. There is increasing evidence that suggest that it is a good immunotherapy target for pediatric brain tumors (70). Indeed, there is a CD276 targeting CAR T-cell immunotherapy phase I clinical trial investigation (BrainChild-03) in pediatric CNS tumors open and currently recruiting (NCT04185038) (21).

The expression of CD47 is known to facilitate immune evasion by tumors by escaping phagocytosis by macrophages (71), and antibody-mediated inhibition of CD47 is a clinically active of area of immunotherapy (72), with Gilead’s Magrolimab being the most clinically advanced (NCT04313881). CD63 is a lysosomal and exosomal-associated membrane protein of the tetraspanin family that is the only known membrane receptor to interact with TIMP-1 an inhibitor of MMPs such as ADAM-10 (73).

Next, we validated the cell surface protein expression of some selected cell surface proteins including CD276, CD63, and CD47. A panel of five DIPG cell lines (SU-DIPG4, SU-DIPG13, SU-DIPG17, SU-DIPG21, and SU-DIPG33) was screened using surface antibody labeling and flow cytometry (**Figures 5B–D**). When compared to the control samples, all SU-DIPG cell lines labeled strongly with antibodies particularly SU-DIPG4 and SU-DIPG13 which had high CD47 expression, whereas SU-DIPG33 had the highest CD276 expression, validating our proteomics findings.

Furthermore, to identify membrane proteins in the dataset, we parsed the 800 shared proteins through the TMHMM server, and, based on a PredHel score of 3 or more, we identified the top 75 proteins (**Supplementary File 7D**). This also included two CD markers (CD47 and CD63) along with several SLC proteins associated with neuronal pathophysiology (74).

## 4 Discussion

DIPG is one of the most aggressive pediatric cancers and remains a near-universally fatal brainstem tumor. Initial studies in DIPG employed genomics and transcriptomics (11, 15, 75) to elucidate disease pathogenesis (11, 12, 15, 75, 76). Only a few studies have explored the proteomic landscape of DIPG using either tissue samples or cerebrospinal fluid of patients (18, 77, 78), and, to our knowledge, there have been no studies elucidating the immunopeptidome or cell surfaceome of DIPG. Related studies in adult GBM and other cancers have indicated that direct analysis of the immunopeptidome of either the primary tumors or patient-derived cell lines aids in identification of tumor-associated peptides and neoepitopes (29, 79, 80).

The study successfully identified 20,536 non-redundant endogenous HLA class I peptides (at 5% FDR) displayed on the surface of six DIPG cell lines, which originated from 6,312 source proteins. Importantly, we identified peptides not only restricted to HLA-A*02:01 but also other high frequency class I alleles, thereby covering 90% of HLA allotypes found in Caucasians (81), of cautionary note when considering designing equitable therapies across ethnicities. The difference in number of HLA class I–restricted peptides identified in SF7761 (12,598) and other SU-DIPG cell lines (despite SU-DIPG58 and SF7761 having similar cell pellet size of 2 × 10^8^) could be attributed to SF7761 being immortalized using human telomerase ribonucleoprotein reverse transcriptase (82). In contrast, SU-DIPG cell lines were cultured directly from post-autopsy tumors.

For a proportion of proteins, there was an association between antigen abundance in the proteomics data and the number of HLA-bound peptides. Expectedly, across all six cell lines, the proteins that contributed to the highest number of peptides (11 or more) are known to be highly abundant in cells including Vimentin, Enolase, Actin, and Elongation factor1. However, for many other antigens such as NLGN4, Abelson Tyrosine-Protein Kinase 1 (ABL), Myelocytomatosis proto oncogene (MYC), v-raf murine sarcoma viral oncogene homolog B1 (BRAF), and Enhancer Of Zeste Homolog 2 (EZH2), one to two peptide ligands were identified despite low levels of protein expression in patient-derived DIPG cell lines. This demonstrated that the immunopeptidome is sampling not only the enriched proteins but also the low abundance ones.

We also compared our DIPG data with the previously published GBM study of Neidert et al. (29), which comprised HLA-restricted peptides identified in glioblastoma stem cell lines, GBM patients’ autopsy samples, and non-diseased PB samples. At the immunopeptidome level, we found an overlap of only 6% (1,240 peptides) and 2% (413 peptides) when compared to GBM patients and PB, respectively. In contrast, at the source protein level, the overlap was considerably higher with 30% (2,034) and 17% (1,134) proteins shared between patient-derived DIPG cell lines and GBM patients and PB, respectively. Of note, there were seven peptides that were demonstrated as being immunogenic in GBM patients in the works of Neidert et al. (29) and Dutoit et al. (56), which were also identified in our study of DIPG.

The data were mined to identify peptides derived from cancer-associated antigens, leading to the identification of 977 TAA- and CTA-derived peptides, of which 234 were HLA-A*02:01–restricted peptides. We found several peptides from proteins known to be dysregulated in DIPG, including IL13Rα2, PRAME, and MAGE, which are also known to generate strong, anti-tumor responses in neuroblastoma and acute myeloid leukemia (83, 84). We also identified 10 HERV peptides originating from PEG10 and PEG3 proteins, which are expressed exclusively in the brain, placenta, and adrenal glands, making these peptides also attractive targets for immunotherapy.

Exploring the proteome can provide insights into disease pathogenesis and progression, and, using this approch, we found enrichment of proteins involved in chromatin remodeling and DNA repair in DIPG cultures. In addtion, we found enrichment of several components of antigen processing and presentation machinery (i.e., HLA-A, TAP, and 26S proteasome machinery) in the DIPG cultures, indicating intact antigen processing and presentation pathways and further validating a T-cell–mediated approach to DIPG treatment. Moreover, in this study, we used two strategies to identify surface proteins expressed in DIPG. A limitation of this study was the lack of availability of PB tissue samples from children, which limited the comparison between DIPG cells lines and healthy brain. Both global proteomics and cell surfaceome enrichment were in strong agreement and identified several novel surface proteins that are potential targets for immunotherapy and/or potential biomarkers. An interesting category of proteins, which was identified using both approaches, included ECM proteins (85) such as integrin, FN1, and TNC proteins whose role is well characterized in GBM (86–88) and only recently has been highlighted in DIPG (89). Intriguingly, ECM proteins are both targets for chemotherapy (90) as well as CAR T cells (91) or oncolytic virotherapy (92, 93). Modulation in levels of proteoglycans such as Glypican-1 and CSPG4 were also observed, in which both were found to be enriched in DIPG cell lines.

Interestingly, the drug targets identified in this study include PARP1, for which there is an active phase II clinical trial testing PARP-inhibitor Veliparib (ABT-888) in combination with radiotherapy and temzolomiode for H3 WT high-grade glioma (NCT03581292). HDAC1 was identified in the total proteomics screen as highly overexpressed in DIPG. Histone deacetylase inhibitors have been the subject of intense study in pediatric brain tumors (94) as candidate drug targets. Panobinostat has previously been found to be a potent agent in a screen of DIPG viability, and knockdown of HDAC1 or HDAC2 also decreased DIPG cell viability (95) and has shown to be brain-penetrant (96). In another study, quisinostat and romidepsin were shown to be effective in DMG mouse models, supporting further exploration (20). We report BChE to be highly abundant in DIPG. Previous studies have shown that high BChE can cause disrupted neurotransmission in Alzheimer’s disease (97); however, it remains unexplored as a target for brain cancer. Similarly, we identified CDK4 to be highly expressed in DIPG, an enzyme important for cell division, which may serve as a therapeutic target for combination strategies (98). CDK4 was, recently, the subject of the pediatric brain tumor consortium study (PBTC-042), in a phase I trial of palboclibin; however, DIPG was excluded (99). Our data also identified translocator protein TSPO, previously shown to also be overexpressed in glioblastoma (100) and worthy of consideration in any future combination therapies. In contrast, CYP51A1, LPL, and TOP1 have no currently known connections with DIPG and may warrant future investigation. The recently published GD2-specific CAR T-cell therapy clinical trial for H2K27M-mutated DIPG/DMG highlighted the promise of an immunotherapy approach to treat DIPG (19). Clearly, there is an incredible opportunity to translate further immunotherapy targets, particularly in multitargeted approaches, but target identification has lacked in the field. Here, we identify several new and potentially promising cell surface targets worthy of further investigation. Some of the proteins identified in this study such as CD276 are already under investigation, both pre-clinically (101, 102) and in humans, with recent studies showing the clinical administration of CD276 (B7-H3)–targeted CAR T cells to treat patients with GBM (103) and an on ongoing phase I clinical trial (21). CD99 has a controversial mechanism of action but is emerging as a novel therapeutic target (104) particularly in malignant glioma (105). Generation of such comprehensive cell surface proteome maps not only will identify new immune-oncology targets but will also facilitate combination approaches, including co-designing therapies to work in conjunction with drug delivery that overcomes the blood–brain barrier.

To summarize, by adopting a multi-pronged approach of HLA-peptide centric antigen discovery, we have, for the first time, identified a comprehensive repertoire of not only potential targetable surface antigens for DIPG immunotherapy but also a range of TAA- and CTA-derived and HERV peptide antigens presented by a number of different HLA allotypes. By considering the surface landscape of DIPG, including both the immune visible HLA-bound peptides along with druggable targets and the protein surfaceome, we will be able to design better tailored combination therapy approaches to treat this devastating disease. Our analysis provides the first comprehensive description of both intracellular-derived and extracellular therapeutic peptide antigen targets for DIPG, which we hope will serve as a valuable tool for the pediatric brain cancer research community.

## Data availability statement

The mass spectrometry immunopeptidomics and whole proteomics data have been submitted to the ProteomeXchange Consortium through the PRIDE partner repository https://proteomecentral.proteomexchange.org/cgi/GetDataset. The dataset identifier for immunopeptidomics is PXD038271 (Project DOI: 10.6019/PXD038271), and for whole proteomics, it is PXD038372.

## Ethics statement

The studies involving human participants were reviewed and approved by WEHI Human Ethics Committee, approval number 34049. Written informed consent to participate in this study was provided by the participants’ legal guardian/next of kin.

## Author contributions

KP, SW, NM, SR, RC, AW, MJ, and AP conceived and planned experiments. KP, SW, JS, and AD conducted experiments. MM provided cell lines and related resources. KP, SR, and AP contributed to immunopeptidomics and proteomics mass spectrometry experiments, analyses, and bioinformatics analyses. KP, NM, SR, RC, MR, and AP wrote the manuscript. SW, RC, MM, and MJ provided feedback. All authors contributed to the article and approved the submitted version.
