# Supplementary material for: A combined immunopeptidomics, proteomics, and cell surface proteomics approach to identify immunotherapy targets for diffuse intrinsic pontine glioma
Source: Front Oncol. 2023 Aug 11;13:1192448. doi: 10.3389/fonc.2023.1192448 (PMC10455951; doi:10.3389/fonc.2023.1192448)
Supplement: Supplementary file 1 [file DataSheet_1.pdf]

## Supplementary Figures and Tables

**Supplementary Table 1.** HLA and immunopeptidomics profiles of DIPG cell lines.

| Cell line | HLA class I typing | HLA-A*02:01 peptides (8-12mer) | HLA-A*03:01 peptides (8-12mer) | Pan HLA class I peptides (8-12mer) | Total HLA class I peptides (8-12mer) |
|---|---|---|---|---|---|
| SU-DIPG27 | A*02:01, A*68:01 B*35:12, C*04:01 | 1,808 | N/A | 1,057 | 2,865 |
| SU-DIPG35 | A*02:01, A*30:01 B*13:02, B*40:01 C*03:04, C*06:02 | 2,671 | N/A | 547 | 3,218 |
| SU-DIPG38 | A*01:01, A*02:01 B*35:03, B*44:02 C*04:01, C*05:01 | 1,418 | N/A | 1,556 | 2,974 |
| SU-DIPG43 | A*02:01, B*44:02, B*45:01 C*05:01 C*16:01 | 1,562 | N/A | 270 | 1,832 |
| SU-DIPG58* | A*01:01, A*03:01 B*07:02, B*08:01 C*07:01, C*07:02 | N/A | 2,115 | 1,530 | 3,645 |
| SF7761* | A*29:02, A*68:01 B*44:03, B*51:02 C*08:01, C*16:01 | N/A | N/A | 11,582 | 11,582 |
| Total | | 7,459 | 2,115 | 16,542 | 26,116 |

* Pellet size 2e8

**Supplementary Table 2.** Published TAAs and brain enriched peptides identified in this study.

| **Peptide sequence** | **HLA restriction** | **Database/ Study** | **Source protein** | **Cancer** |
|---|---|---|---|---|
| LLDRFLATV | A2 | TANTIGEN | Cyclin-1 (CCNI) | Ovarian ≠ |
| ILDDIGHGV | A2 | TANTIGEN | Abl interactor 2 (ABI2) | Ovarian ≠ |
| KLDVGNAEV | A2 | TANTIGEN | B-cell receptor-associated protein 31 (BAP31) | Ovarian ≠ |
| KLQELNYNL | A2 | TANTIGEN | Signal transducer and activator of transcription 1(STAT1) | Ovarian ≠ |
| RLYPWGVVEV | A2 | TANTIGEN | Septin-2 (SEPT2) | Ovarian ≠ |
| FLYDDNQRV | A2 | TANTIGEN | DNA topoisomerase 2-alpha (TOP2A) | Ovarian ≠ |
| SLLQHLIGL | A2 | TANTIGEN | Preferentially expressed antigen of melanoma (PRAME) | Ovarian ≠ |
| KVLEYVIKV | A2 | TANTIGEN | Melanoma-associated antigen 1 (MAGA1) | Ovarian ≠ |
| FLDPRPLTV | A2 | TANTIGEN | Cytochrome P450 1B1 (CP1B1) | Ovarian ≠ |
| GLAPPQHLIRV | A2 | TANTIGEN | TP53 | |
| ETIPLTAEKL | A*68:01 | TANTIGEN | Cyclin-D1 (CCND1) | Renal^ |
| VGLIRNLAL | B8 | TANTIGEN | Beta-catenin (CTNB1) | Renal^ |
| KIQEILTQV | A2 | TANTIGEN Dutoit *et al* | Insulin Growth factor 2 binding protein (IGF2BP3) | GBM |
| TMLARLASA | A2 | TANTIGEN Dutoit *et al* | Chondroitin sulphate proteoglycan 4 (CSPG4) | GBM |
| NLDTLMTYV | A2 | HBPA Dutoit *et al* | Neuroligin 4, X-linked (NLGN4X) | GBM |
| AIIDGVESV | A2 | HBPA Dutoit *et al* | Protein tyrosine phosphatase, receptor-type Z polypeptide 1 (PTPRZ1) | GBM |
| KVFAGIPTV | A2 | HBPA Dutoit *et al* | PTPRZ1 | GBM |
| SILDIVTKV | A2 | HBPA Neidert *et al* | (RFTN2) | GBM |
| LTFGDVVAVR | A*68:01 | HBPA Dutoit *et al* (LTFGDVVAV) | Fatty acid-binding protein 7, brain (FABP7) | GBM |

^ **(Weinschenk et al. 2002)**

**≠ (Ramakrishna et al. 2003)**

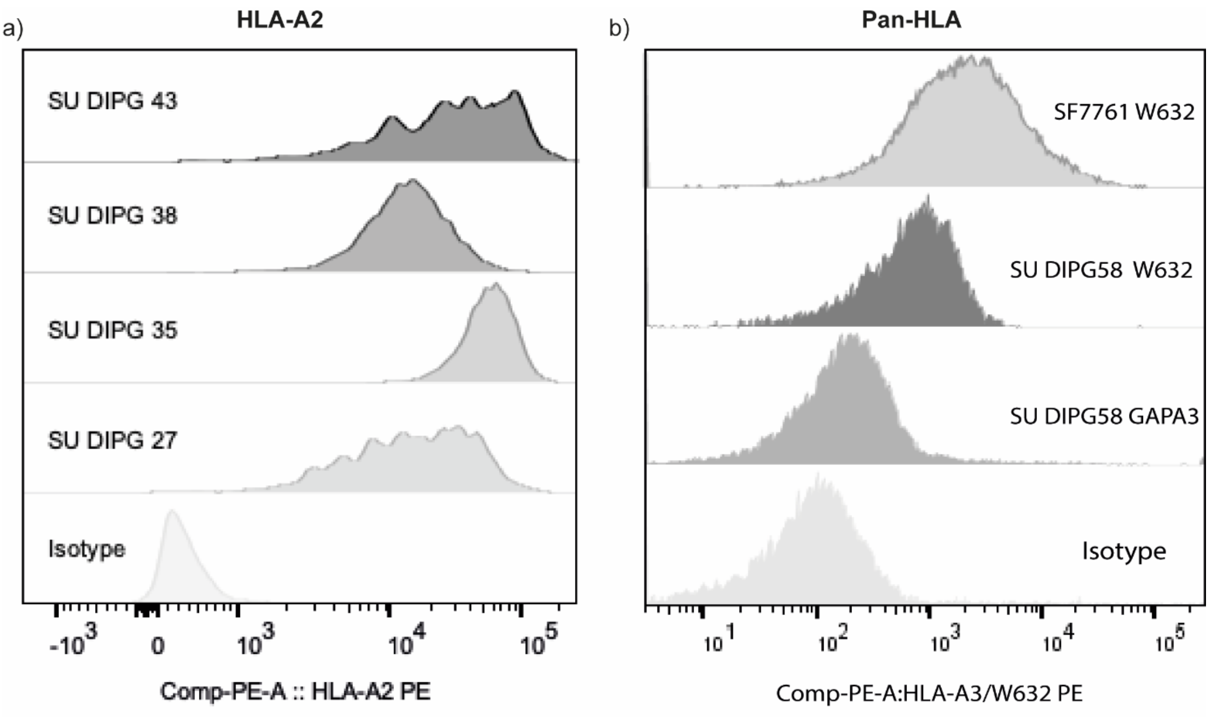


**Supplementary Figure 1 - HLA class I cell surface expression on DIPG cell lines SU-DIPG27, SU-DIPG35, SU-DIPG38, SU-DIPG43, SU-DIPG58 and SF7761.** Cells were stained with either **a)** BB7.2 (anti-HLA-A2), **b)** GAPA3 (anti-HLA-A3) or W6/32 (pan HLA class I) antibodies followed by secondary antibody ($2^{nd}$; goat anti-mouse IgG PE). Cells were acquired on LSR IIa (BD Biosciences) using the FACSDiva software. Analysis of the cell population was performed using FlowJo software (BD Biosciences, USA.)

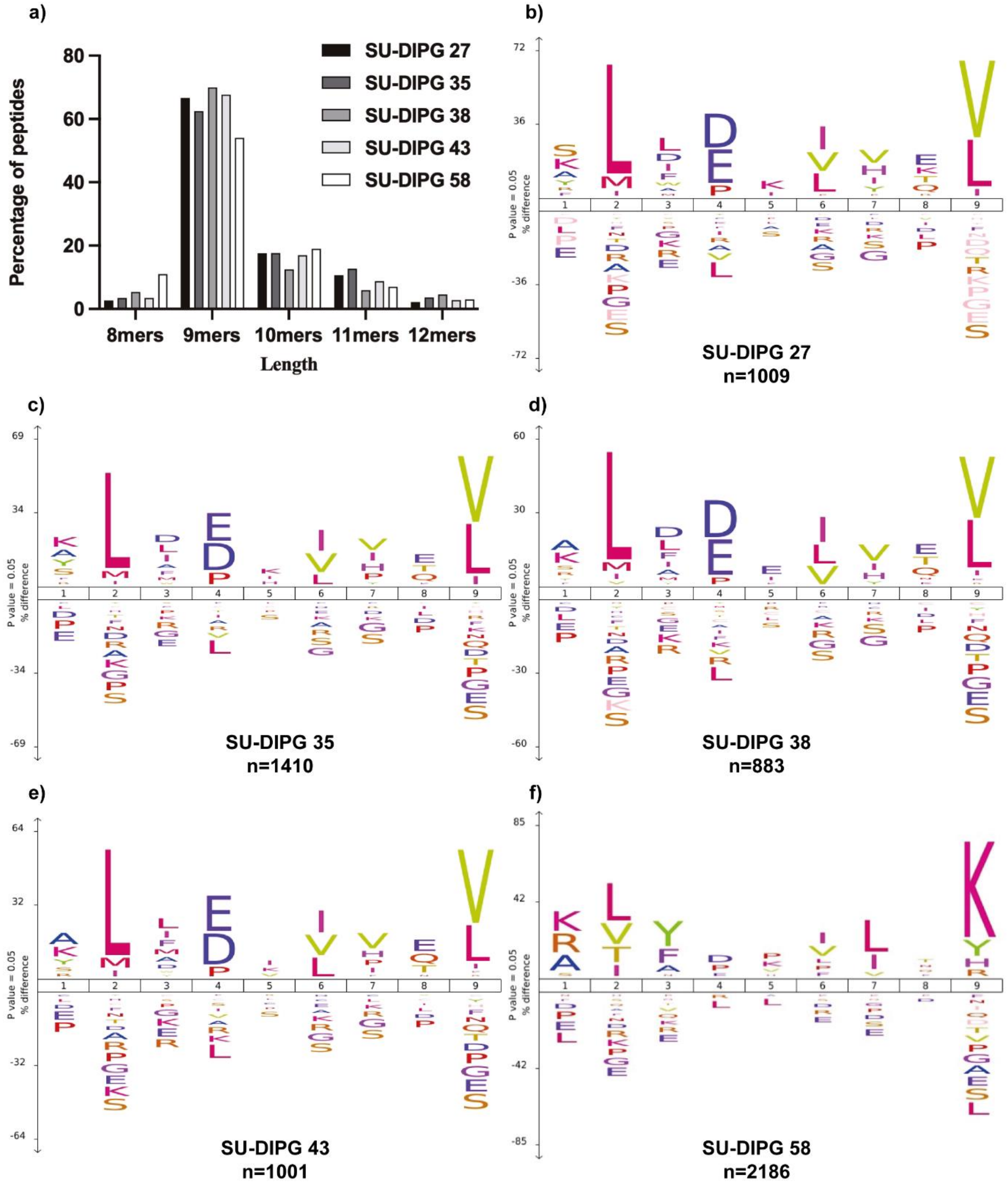


**Supplementary Figure 2 – HLA-A*02:01 and A*03:01 derived DIPG peptides follow canonical peptide length distribution and consensus binding motif.** a) The majority of peptides identified in each cell line were nonamers. HLA-binding motifs of 9mer peptides identified in b) SU-DIPG27, c) SU-DIPG35, d) SU-DIPG38 e) SU-DIPG43 and f) SU-DIPG58 plotted using IceLogo (Colaert et al., 2009) follow canonical HLA-A2 and HLA-A3 motif with n depicting the number of peptides, samples acquired as n=1 biological replicate.

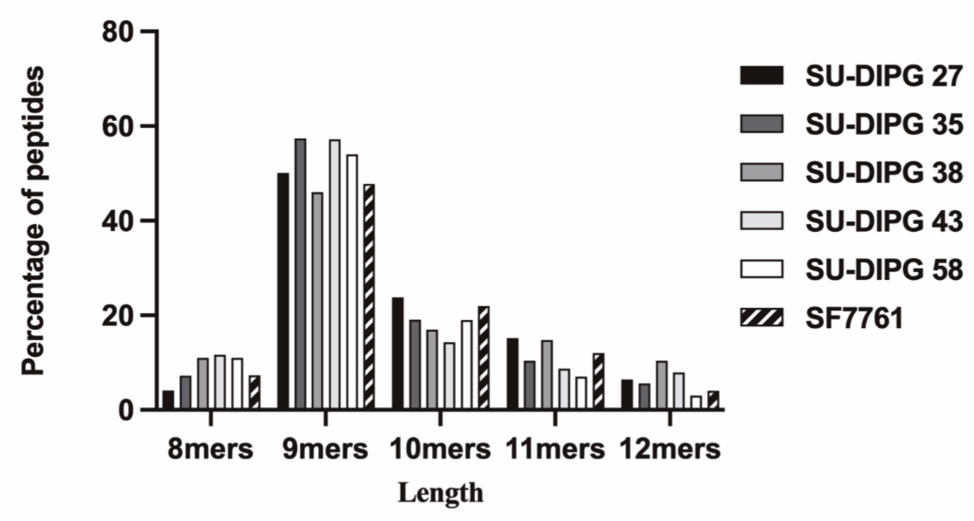


**Supplementary Figure 3 – Pan HLA class I-derived DIPG peptides follow canonical peptide length distribution.** The majority of peptides identified in each DIPG cell line were nonamers. Samples acquired as n=1 biological replicate.

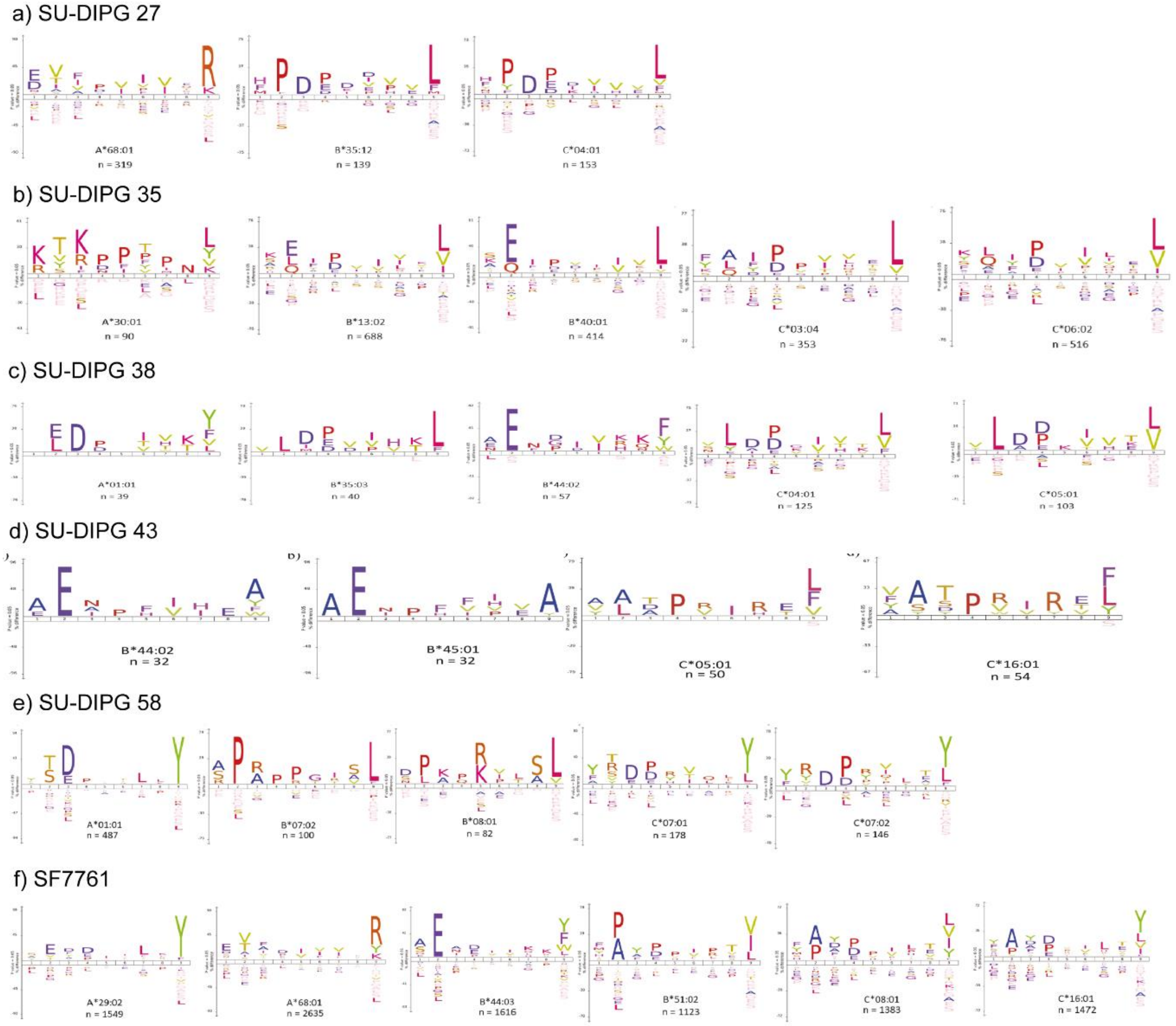


**Supplementary Figure 4 – DIPG cell lines show canonical HLA class I binding motifs. a)** SU-DIPG27, **b)** SU-DIPG35, **c)** SU-DIPG38, **d)** SU-DIPG43 and **e)** SU-DIPG58 and **f)** SF7761. Nonamers were selected, with modifications and duplicates being removed. Peptide binding affinity was predicted using NetMHC Pan 4.0. Icelogo (Colaert et al., 2009), with n representing number of binders identified for each allele.

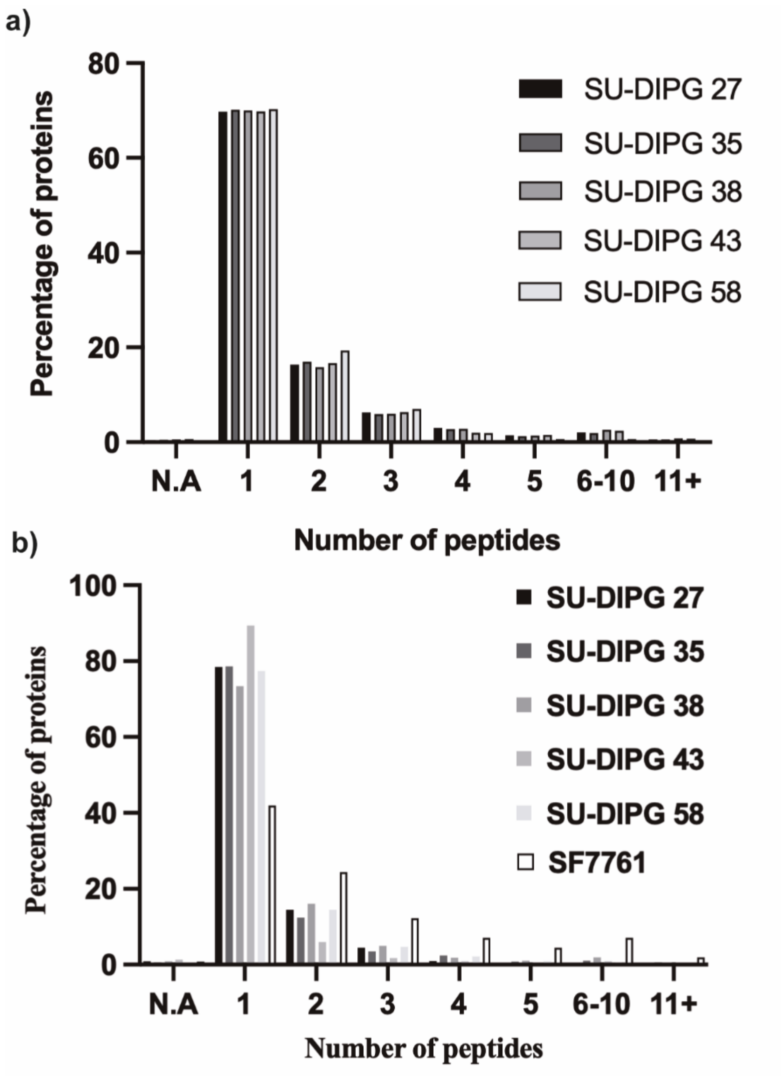


**Supplementary Figure 5 – Histogram depicting number of HLA class I peptides presented by different source proteins identified across DIPG cell lines using a)** HLA specific antibodies anti-A2 (BB7.2) and anti-A3 (GAPA3) **b)** Anti-Pan class I antibody (W6/32). Majority of peptides (70-85%) regardless of antibodies used for immunopeptidomics came from only 1 protein. N.A – is non-assigned

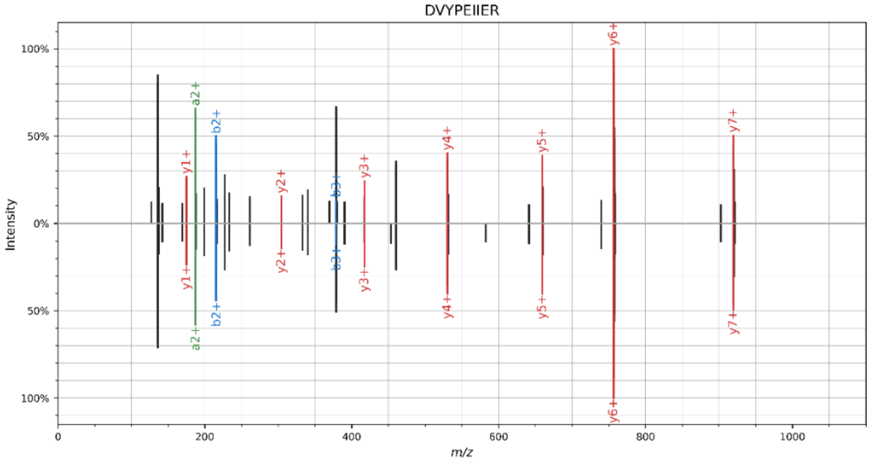
DVYPEIIER
Intensity
m/z
100%
50%
0%
50%
100%
0
200
400
600
800
1000
y1+
a2+
b2+
y2+
b3+
y3+
y4+
y5+
y6+
y7+

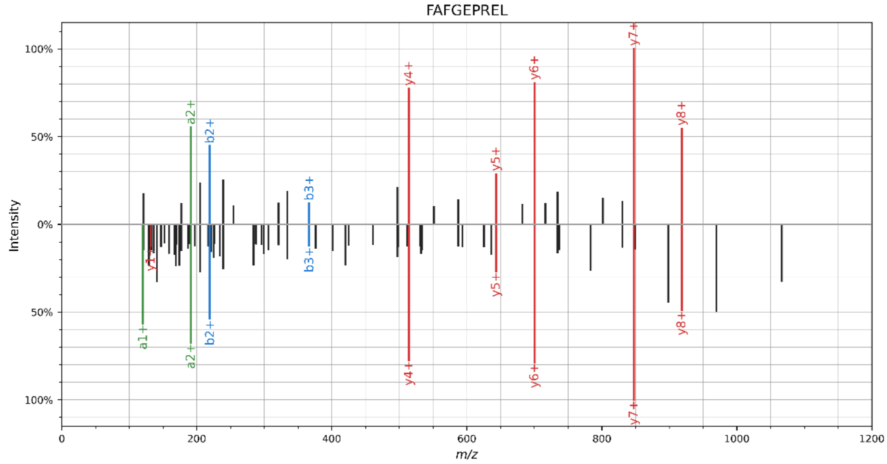
FAFGEPREL
Intensity
m/z
100%
50%
0%
50%
100%
0
200
400
600
800
1000
1200
a1+
y1+
a2+
b2+
b3+
y4+
y5+
y6+
y7+
y8+

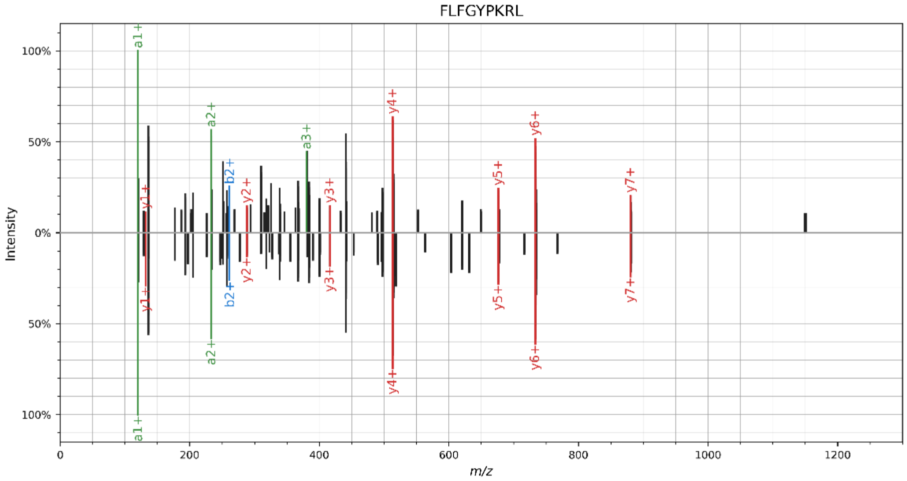
FLFGYPKRL
Intensity
m/z
100%
50%
0%
50%
100%
0
200
400
600
800
1000
1200
a1+
y1+
a2+
b2+
y2+
a3+
y3+
y4+
y5+
y6+
y7+

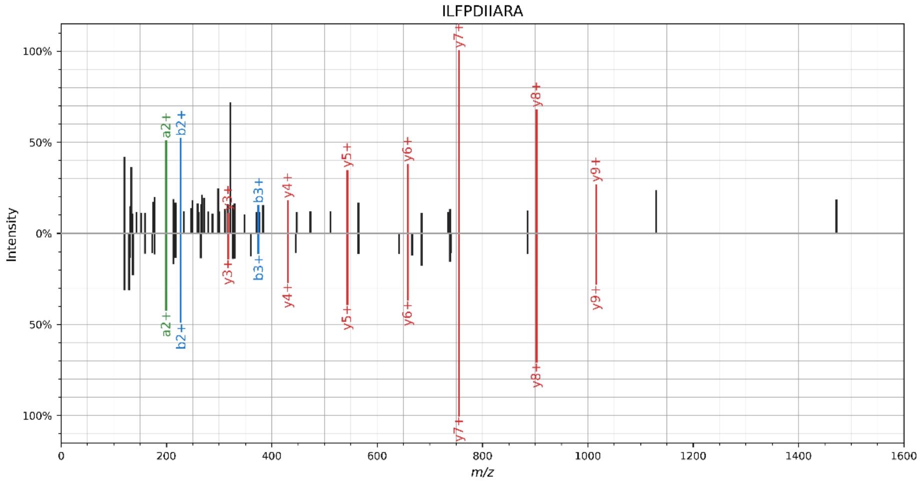
ILFPDIIARA
Intensity
m/z
100%
50%
0%
50%
100%
0
200
400
600
800
1000
1200
1400
1600
a2+
b2+
y3+
b3+
y4+
y5+
y6+
y7+
y8+
y9+

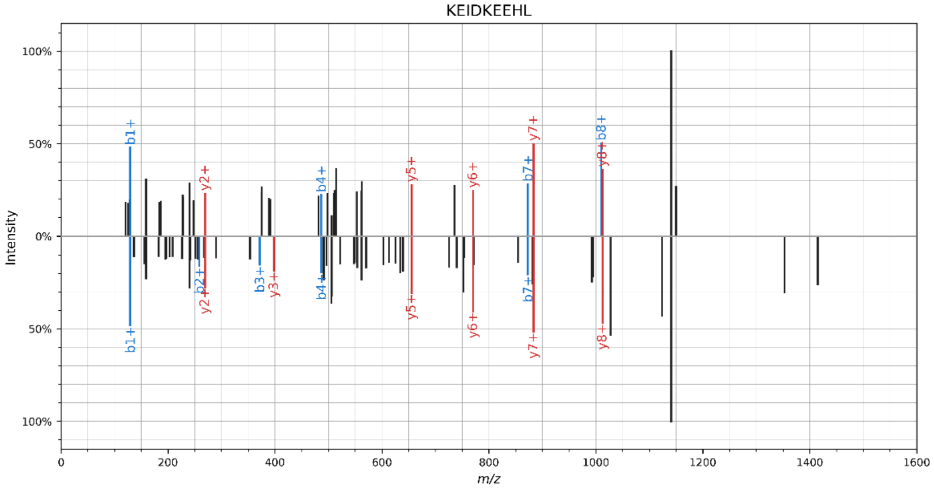
KEIDKEEHL
Intensity
m/z
100%
50%
0%
50%
100%
0
200
400
600
800
1000
1200
1400
1600
b1+
y2+
b2+
b3+
y3+
b4+
y5+
y6+
b7+
y7+
b8+
y8+

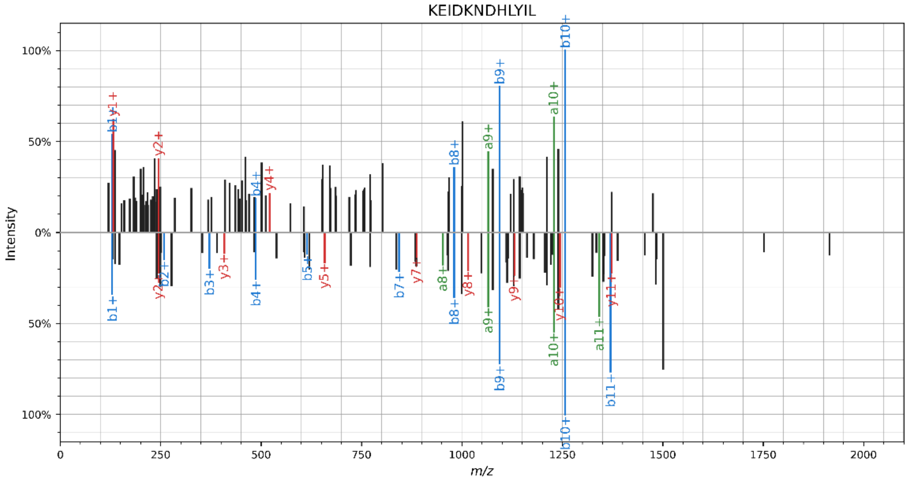
KEIDKNDHLYIL
Intensity
m/z
100%
50%
0%
50%
100%
0
250
500
750
1000
1250
1500
1750
2000
b1+
y1+
y2+
b2+
b3+
y3+
b4+
y4+
b5+
y5+
b7+
y7+
a8+
b8+
y8+
a9+
b9+
y9+
a10+
b10+
y10+
a11+
b11+
y11+

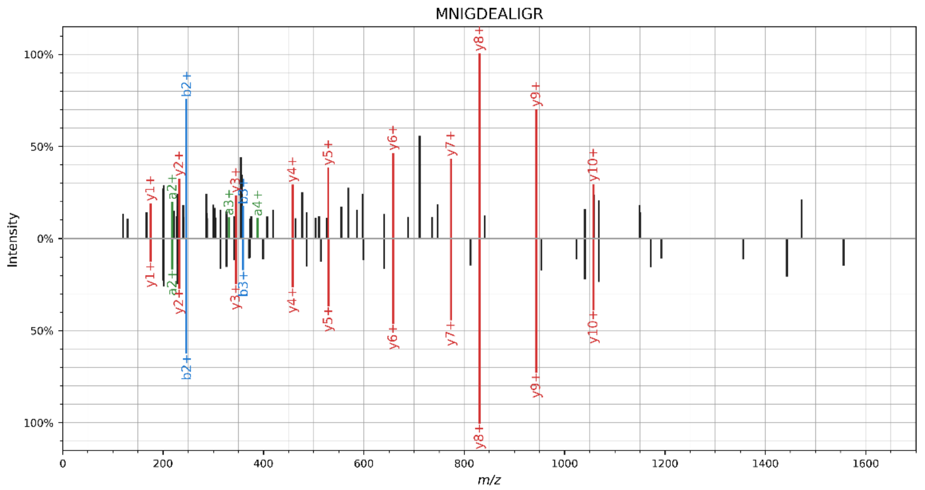
MNIGDEALIGR
Intensity
m/z

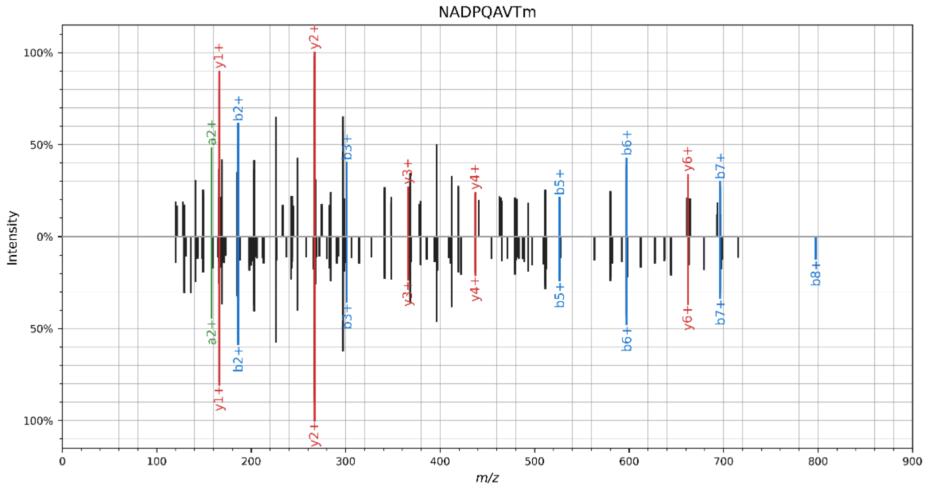
NADPQAVTm
Intensity
m/z

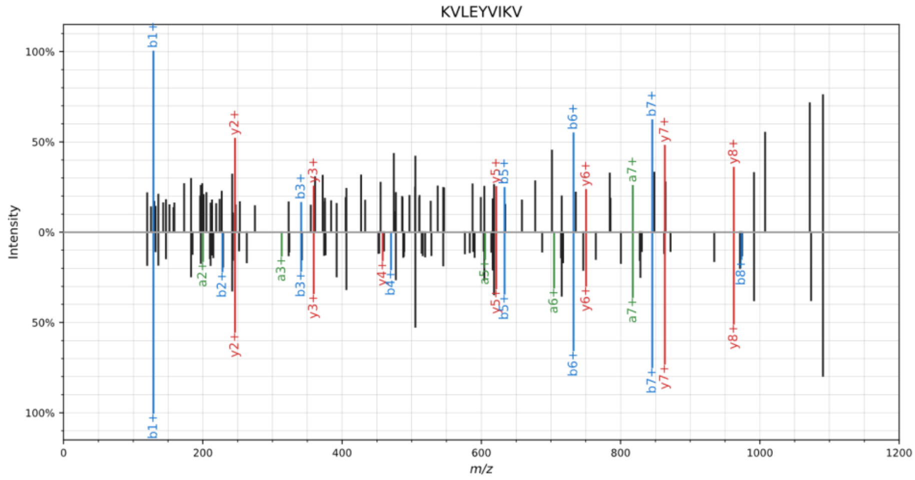
KVLEYVIKV
Intensity
m/z

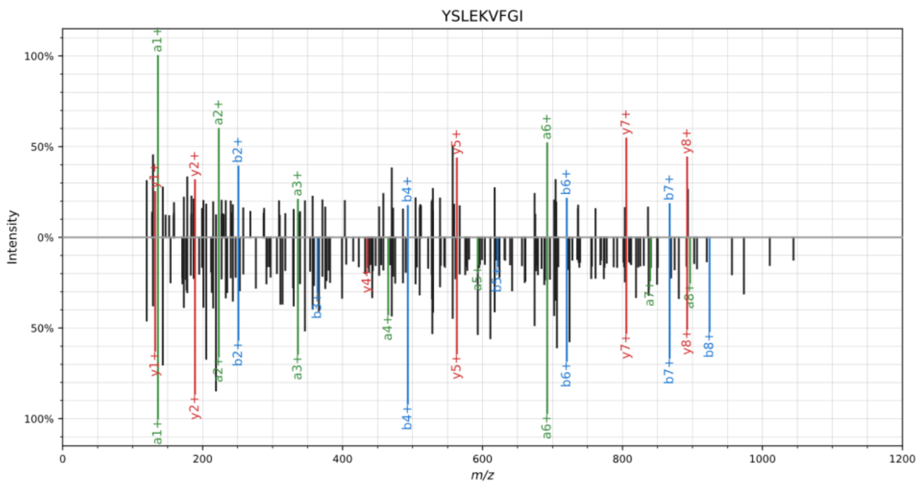


**Supplementary Figure 6 - Annotated mirror plots of 10 CTA peptides** depicting experimental (top) and synthetic (bottom) spectra with highlighted b and y ions identified from

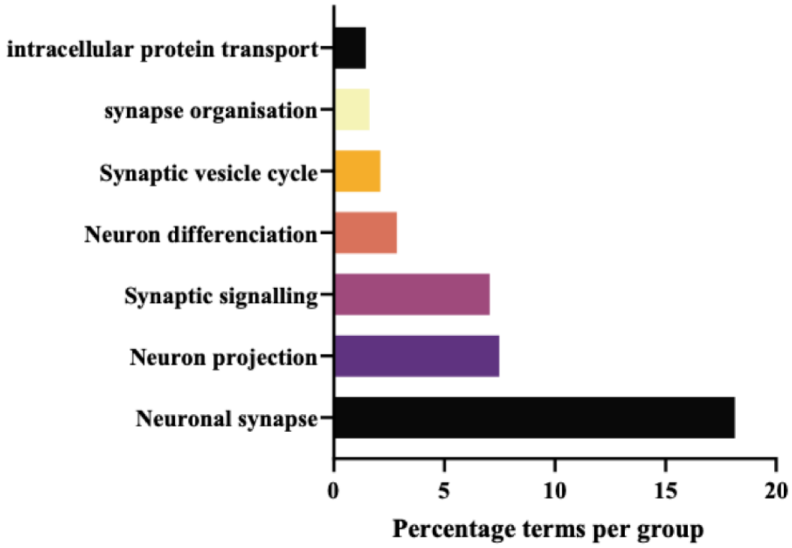


**Supplementary Figure 7 – Common proteins between DIPG cell lines and primary brain tissue participate in neuronal and synapse pathways**. Pathway analysis using GO terms in Clue GO (Cytoscape) for the overlapping proteins revealed they are involved in neuron and synapse cross talk.

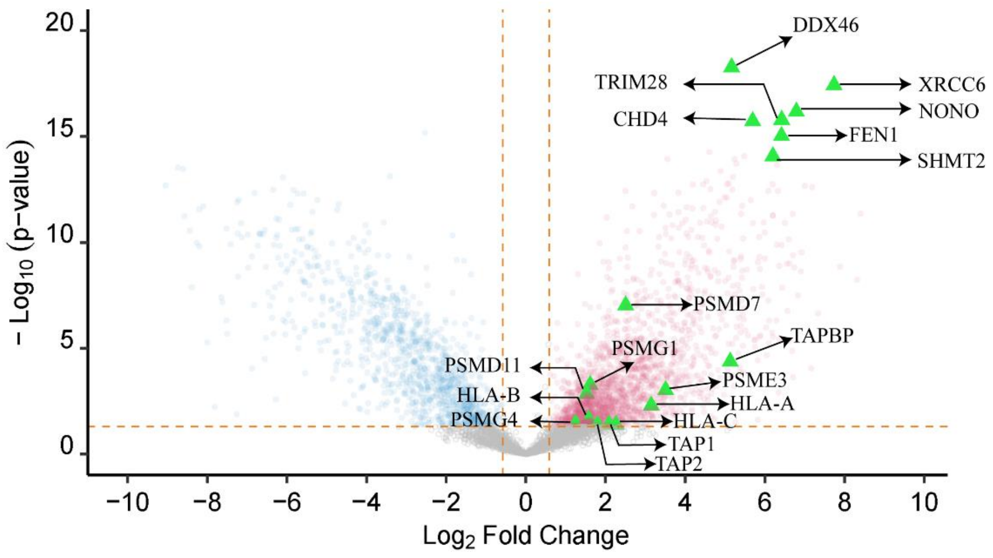


**Supplementary Figure 8 – A comparison of the proteomic landscape of DIPG cell lines and primary brain samples.** Volcano plot depicting log2 fold change and corresponding -log10 adjusted p-value of all differentially expressed proteins in DIPG cell lines when compared to primary brain controls, with more abundant proteins (pink), less abundant proteins (steel blue) and proteins with no significant difference in relative abundance (grey) shown. Proteins of interest are denoted in green triangles.
